# Haematological malignancies in relatives of patients affected with myeloproliferative neoplasms

**DOI:** 10.1002/jha2.425

**Published:** 2022-03-24

**Authors:** Daniele Vanni, Oscar Borsani, Yasuhito Nannya, Emanuela Sant'Antonio, Chiara Trotti, Ilaria Carola Casetti, Daniela Pietra, Anna Gallì, Silvia Zibellini, Virginia Valeria Ferretti, Luca Malcovati, Seishi Ogawa, Luca Arcaini, Elisa Rumi

**Affiliations:** ^1^ Department of Molecular Medicine University of Pavia Pavia Italy; ^2^ Division of Haematology Fondazione Istituto di Ricovero e Cura a Carattere Scientifico (IRCCS) Policlinico San Matteo Pavia Italy; ^3^ Department of Pathology and Tumor Biology Kyoto University Kyoto Japan; ^4^ Division of Hematopoietic Disease Control The Institute of Medical Sciences The University of Tokyo Tokyo Japan; ^5^ Division of Haematology Azienda USL Toscana Nord Ovest Lucca Italy; ^6^ Service of Clinical Epidemiology and Biostatistics Fondazione Istituto di Ricovero e Cura a Carattere Scientifico (IRCCS) Policlinico San Matteo Pavia Italy

**Keywords:** familial, malignancies, myeloproliferative, relatives

## Abstract

In a cohort of 3131 patients with myeloproliferative neoplasms (MPNs), we identified 200 patients (6.4%) who reported a second case of haematological malignancies (HM) in first‐ or second‐degree relatives. The occurrence of a second HM in the family was not influenced by MPN subtype, sex or driver mutation, while it was associated with age at MPN diagnosis: 8.5% of patients diagnosed with MPN younger than 45 years had a second relative affected with HM compared to 5.5% of those diagnosed at the age of 45 years or older (*p = *0.003), thus suggesting a genetic predisposition to HM with early onset.

## 1 INTRODUCTION

Myeloproliferative neoplasms (MPNs) are a heterogenous group of clonal diseases, characterized by an increased production of differentiated haematopoietic cells, that occur mostly in a sporadic way [1]; however, a non‐trivial proportion of cases (7.6%) are, indeed, familial [2, 3]. In this regard, patients’ relatives have a five to seven fold higher risk of developing MPN compared to the general population [4]. This notion has been recently confirmed and extended to other subtypes of myeloid neoplasms, thanks to two large, population‐based studies that focused on familial aggregation of haematological neoplasms [5, 6]. Even though first‐degree relatives had the strongest relative risk for the same disease showed by the index case, a more general, increased risk of all myeloid malignancies emerged, that correlated with the number of affected relatives and, especially for MPN, with younger age of the index case [5]. Moreover, there was evidence for pleiotropic associations among lymphoid and myeloid malignancies [4], supporting the hypothesis of a shared aetiology [7], either inherited [8] or environmental [9, 10]. In the present study, we aimed at estimating risk of haematological malignancies (HMs) among relatives of MPN patients and performed whole exome sequencing (WES) on four patients from two representative families.

### 1.1 Patients and methods

### 1.2 Patients

We interrogated our database of MPN patients, followed at our institution from 1970 to 2020, to identify patients that reported at least a second case of HM in first‐ or second‐degree relatives. Medical records of relatives were reviewed when available. In our routine clinical practice, we routinely interview all patients referred to, or diagnosed with MPN, at our Institution for other haematological cancer diagnoses among their relatives, both at the time of first referral and, regularly, during their follow‐up.

## 2 METHODS

DNA for driver mutation analysis was available in 2392 of 3131 patients. *JAK2* V617F mutation, *CALR* exon 9 mutations and *MPL* exon 10 mutations were assessed as previously described [11].

**FIGURE 1:**
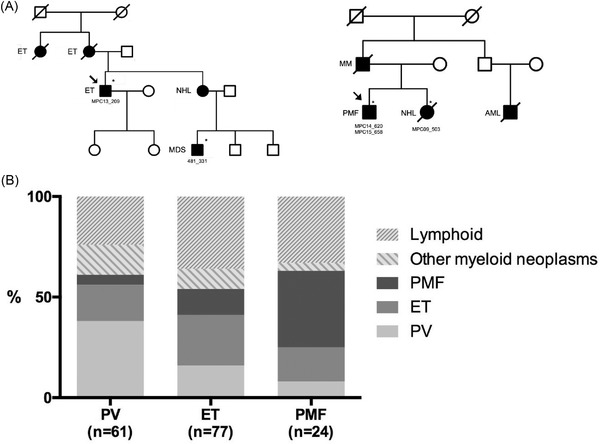
(A) Pedigrees of two representative families with multiple cases of haematological malignancies. Pedigrees of family #126 (left) and family #127 (right) are drawn. Filled symbols represent affected members, slashes indicate deceased members, stars indicate members with available DNA and arrows indicate the proband. DNA of healthy relatives was not available. AML, acute myeloid leukaemia; ET, essential thrombocythemia; MDS, myelodysplastic syndrome; MM, multiple myeloma; NHL, non‐Hodgkin lymphoma; PMF, primary myelofibrosis. (B) Graphical representation showing the distribution of haematological diagnosis of relatives of 162 myeloproliferative neoplasm (MPN) patients according with the diagnosis of the index case. Columns represent the percentage of relatives affected with polycythemia vera (PV), essential thrombocythemia (ET), primary myelofibrosis (PMF), other myeloid neoplasms and lymphoid neoplasms according to the diagnosis of the index case (PV, ET or PMF). ‘Lymphoid’ category comprises the following entities: B‐cell non‐Hodgkin lymphoma, Hodgkin lymphoma, B‐lymphoblastic leukaemia/lymphoma, chronic lymphocytic leukaemia, plasma cell neoplasm and T‐cell lymphoma. ‘Other myeloid neoplasms’ category comprises the following: acute myeloid leukaemia, chronic myeloid leukaemia, mastocytosis and myelodysplastic syndrome. Four patients (one diagnosed with MPN‐unclassifiable and three with relatives diagnosed with MPN‐unclassifiable) are not included in the graph for practical purposes

In two representative families with several cases of HM (family #126 and family #127 reported in Figure 1A), WES was performed in two cases of each family. WES libraries were prepared using xGen Exome Research Panel integrated DNA technologies, followed by sequencing of enriched fragments on a Novaseq 6000 system (Illumina) in 150 bp paired‐end mode. The target depth was 100×, and the actual depth was 138× (129–158×). Mutation calling was performed using the Genomon2 pipeline (v.2.6), as previously described [12]. Significance of mutations was evaluated by the EBCall algorithm [13], on the basis of an empirical distribution of variant allele frequencies (VAFs) as determined using WES data of non‐paired peripheral blood samples (*n* = 20). Somatic mutations in polymorphonuclear cells were examined by using mononuclear cells (for patient 481_331) or sorted T lymphocytes (for the remaining other samples) obtained from the same blood samples as normal controls. Putative germline variants were extracted using non‐controlled analysis where all the non‐synonymous variants in the coding regions or splicing sites in comparison with hg19 reference genome were selected, from which common SNPs (>0.1% in either of 1000g2014oct_all or ExAC.r0.3.1) were filtered out. We selected those variants that were classified as ‘Pathogenic’ or ‘Likely Pathogenic’ according to Clinvar version 20210501 and InterVar version 20180118. We rescued any variants that had been reported to be involved in myeloproliferative phenotype.

Univariate and multivariate analyses were performed to evaluate patient's characteristics associated with an increased familial risk.

Quantitative variables have been summarized as median and interquartile range. Qualitative variables were described as counts and percentages of each category and are reported together with the exact binomial 95% confidence intervals.

Associations between two qualitative variables were tested via Fisher's exact test. Mann–Whitney test was used to compare quantitative variables between two independent groups of patients, while Wilcoxon matched‐pairs signed‐rank test was applied to compare quantitative variables between two paired groups of patients. *p*‐Values lower than 0.05 were considered significant.

All statistical analyses were performed with Stata 16 software (release 16, StataCorp, College Station, TX, USA).

## 3 RESULTS

Within a cohort of 3131 consecutive patients with MPN, we identified 200 patients (6.4%) who reported a second case of HMs in the same family. In 142 of 3131 patients (4.5%), the second case was reported in a first‐degree relative. The occurrence of a second haematological cancer in the family was not influenced by MPN subtype (*p* = 0.527), sex (*p* = 0.079) or driver mutation (*p* = 0.718), while it was associated with age at MPN diagnosis (Table 1): 8.5% of patients diagnosed with MPN younger than 45 years had a second relative affected with HM compared to 5.5% of those diagnosed at the age of 45 years or older (*p = *0.003). Median age at diagnosis of patients with affected relatives was lower than that of patients without affected relatives (51 years vs. 55 years, *p* < 0.001).

**TABLE 1:** Association between characteristics at diagnosis and familial risk

Variables	Patients with affected relatives, *n* (%)	*p*‐Value
**Type of MPN**		0.527
ET	90/1403 (6.4)	
PV	77/1106 (7.0)	
PMF	32/553 (5.8)	
MPN nos	1/69 (1.4)	
**Sex**		0.079
Male	85/1525 (5.6)	
Female	115/1606 (7.2)	
**Age at diagnosis**		0.003
<45 years	76/894 (8.5)	
≥45 years	124/2237 (5.5)	
**Mutational status** *		0.718
*JAK2* V617F	141/1795 (7.9)	
*CALR*	28/401 (7.0)	
*MPL*	4/64 (6.3)	
Triple negative	13/132 (9.9)	

* DNA to assess mutational status was available in 2392 of 3131 patients.

Abbreviations: ET, essential thrombocythemia; MPNs, myeloproliferative neoplasms; PMF, primary myelofibrosis; PV, polycythemia vera.

Among cases with complete available information (166 of 200), 114/166 (68.7%) had a relative with myeloid malignancies, mostly MPN (101 cases), while 52/166 (31.3%), had a relative with lymphoid malignancies. The distribution of diagnoses observed in affected relatives is reported in Figure 1B and in Table S1. Among relatives with HM, the percentage of those affected with MPN was higher than the percentage of those affected with a myeloid disease other than MPN or a lymphoid disorder (60.8%, confidence interval [CI] 53.0%–68.3%, vs. 39.2%, CI 31.7%–47.0%). In MPN families, we confirmed that second generation patients are younger than first generation ones, both as index cases (36 years vs. 47 years, *p* < 0.001) or as relatives (39 years vs. 63 years, *p = *0.007).

Results of WES performed in the two representative families with several cases of HM are reported in Tables S2–S5. The number of putative germline variants with VAF values >0.35 detected in both individuals of each family was 65 in family #126 (Table S2) and 145 in family #127 (Table S3). None of these variants are known to be responsible for MPN phenotype. We also explored the structural variant (SV) calls that are invariably detected in both affected cases of each family and found 7 SV in family #126 (Table S4) and 7 SV in family #127 (Table S5).

Two affected genes were shared by all four members of both families, namely single nucleotide variants in AHNAK2 (c.G14026C, p.D4676H in family 126 and c.G4063C, p.A1355P in family 127) and a same 240 nt‐deletion in CLCN7 (chr16:1500694‐1500933del). However, these abnormalities are unlikely pathogenetic because AHNAK2 substitutions are located in a simple repeat region, and CLCN7 deletion is located within an intron and is unlikely to affect the coding region coordinates. Also, when considering the shared protein family the corresponding proteins belong to, the affected genes are unlikely to have a role in the pathogenesis of MPN disease.

## 4 DISCUSSION

Our findings, in a well‐annotated, large, monocentric cohort of MPN patients, confirm a high familial risk of HM, with a striking prevalence for myeloid neoplasms. It has been previously recognized that MPN patients have an increased risk of developing second primary malignancies [14, 15]. Our observations are in line with two previous papers that identified familial associations of cancer diseases that extended across different hematopoietic cell lineages [6, 7].

Consistent with the notion that patients diagnosed with cancer at a younger age are more likely to have a genetic predisposition [7], frequency of HM among patients’ relatives was linked to age at MPN diagnosis of the index case.

We acknowledge that our study has intrinsic limitations due to the retrospective design and due to a potential risk of missing data or under‐reported diagnoses because family members were not further followed after death of the proband. As a consequence, we could speculate that we have preferentially collected information on families with cancer diagnosed in older individuals (i.e., grandparents and/or parents, when a son is the index MPN case) compared to families with two affected siblings.

In conclusion, our findings support the hypothesis of a significant familial aggregation of HM and underline the importance of pursuing deep‐sequencing approaches in accurately selected families. Unfortunately, in our representative families we did not find the pathogenetic event but we hope that an improved knowledge would be clinically relevant, since it could improve both management and counselling of patients with haematological neoplasms and their relatives.

## CONFLICT OF INTEREST

The authors declare no conflict of interest.

## ETHICS STATEMENT

The study was approved by the Institutional Review Board and the procedures followed were in accordance with the Helsinki Declaration.

## AUTHOR CONTRIBUTIONS

Daniele Vanni, Oscar Borsani, Emanuela Sant'Antonio and and Elisa Rumi conceived the study and wrote the manuscript. Yasuhito Nannya, Daniela Pietra, Anna Gallì and Silvia Zibellini performed molecular analyses and interpreted variants. Daniele Vanni, Oscar Borsani, Chiara Trotti and and Ilaria Carola Casetti collected clinical data. Virginia Valeria Ferretti performed statistical analysis. Luca Malcovati, Seishi Ogawa and Luca Arcaini finalized the manuscript.

## Supporting information

Supporting Information

Supporting Information

Supporting Information

Supporting Information

Supporting Information
