# Supplementary material for: Haematological malignancies in relatives of patients affected with myeloproliferative neoplasms
Source: EJHaem. 2022 Mar 24;3(2):475–9. doi: 10.1002/jha2.425 (PMC9176120; doi:10.1002/jha2.425)
Supplement: Supplementary file 1 — Supporting Information [file JHA2-3-475-s005.docx]

Supplementary table 1 – Detailed list of the 166 MPN patients with at least one relative diagnosed with hematological neoplasm. Patients are ordered first by their MPN diagnostic category and, secondly, according to the hematological diagnosis of the affected relative. Mutational profile was not available for 3 patients (S13A78SM, S34A85SM, S79A10SM). AML, acute myeloid leukemia; B-ALL, B-lymphoblastic leukemia/lymphoma; B-NHL, B-cell non-Hodgkin lymphoma; CLL, chronic lymphocytic leukemia; CML, chronic myeloid leukemia; ET, essential thrombocythemia; HL, Hodgkin lymphoma; MDS, myelodysplastic syndrome; MPN-U, myeloproliferative neoplasm, unclassifiable; PC neoplasm, plasma cell neoplasm; PMF, primary myelofibrosis; PV, polycythemia vera; TCL, T-cell lymphoma

| **Index case** | | | | | **Relatives** | |
| --- | --- | --- | --- | --- | --- | --- |
| **ID** | **Sex** | **Age at diagnosis** | **Diagnosis** | **Mutated gene** | **Kinship with index case** | **Diagnosis** |
| S46A11SM | M | 44 | PV | *JAK2 V617F* | daughter | AML |
| S6A19V | F | 42 | PV | *JAK2 V617F* | uncle | AML |
| S124A15V | M | 57 | PV | *JAK2 V617F* | sister | B-NHL |
| S125A16V | M | 68 | PV | *JAK2 V617F* | brother | B-NHL |
| S131A12V | M | 40 | PV | *JAK2 V617F* | mother | B-NHL |
| S16A12V | M | 66 | PV | *JAK2 V617F* | mother | B-NHL |
| S20A17V | F | 71 | PV | *JAK2 V617F* | son | B-NHL |
| S69A6V | F | 54 | PV | *JAK2 V617F* | brother | B-NHL |
| S71A11V | F | 47 | PV | *JAK2 V617F* | aunt | B-NHL |
| S118A15V | F | 50 | PV | *JAK2 V617F* | cousin | CML |
| S164A8SM | M | 51 | PV | *JAK2 V617F* | mother | CML |
| S40A1V | F | 38 | PV | *JAK2 V617F* | cousin | CML |
| S7A3V | F | 60 | PV | *JAK2 V617F* | brother | CML |
| S103A16V | F | 79 | PV | *JAK2 V617F* | sister | ET |
| S11A11V | F | 57 | PV | *JAK2 V617F* | grand daughter | ET |
| S147A6SM | F | 57 | PV | *JAK2 V617F* | cousin | ET |
| S205A14SM | M | 19 | PV | *JAK2 V617F* | cousin | ET |
| S25A12V | F | 63 | PV | *JAK2 V617F* | sister | ET |
| S30A2V | M | 32 | PV | *JAK2 V617F* | mother | ET |
| S30A7V | M | 52 | PV | *JAK2 V617F* | mother | ET |
| S3A85V | M | 41 | PV | *JAK2 V617F* | aunt | ET |
| S4A10V | F | 42 | PV | *JAK2 V617F* | brother | ET |
| S87A10V | M | 66 | PV | *JAK2 V617F* | daughter | ET |
| S92A6V | F | 70 | PV | *JAK2 V617F* | cousin | ET |
| S25A11V | M | 33 | PV | *JAK2 V617F* | mother | HL |
| S31A9V | F | 55 | PV | *JAK2 V617F* | brother | HL |
| S52A2V | F | 56 | PV | *JAK2 V617F* | grand daughter | HL |
| S10A7V | M | 51 | PV | *JAK2 V617F* | mother | MDS |
| S13A16V | M | 45 | PV | *JAK2 V617F* | aunt | MDS |
| S43A12V | F | 43 | PV | *JAK2 V617F* | mother | MDS |
| S101A7V | M | 60 | PV | *JAK2 V617F* | brother | PC neoplasm |
| S163A14SM | F | 62 | PV | *JAK2 V617F* | mother | PC neoplasm |
| S17A99V | M | 35 | PV | *JAK2 V617F* | father | PC neoplasm |
| S78A12V | M | 59 | PV | *JAK2 V617F* | sister | PC neoplasm |
| S93A16V | F | 57 | PV | *JAK2 V617F* | mother | PC neoplasm |
| S1A0V | F | 72 | PV | *JAK2 V617F* | grand daughter | PMF |
| S67A4V | M | 48 | PV | *JAK2 V617F* | uncle | PMF |
| S79A14V | F | 51 | PV | *JAK2 V617F* | mother | PMF |
| S101A16V | M | 52 | PV | *JAK2 V617F* | sister | PV |
| S110A15V | M | 41 | PV | *JAK2 V617F* | mother | PV |
| S128A10V | M | 60 | PV | *JAK2 V617F* | mother | PV |
| S12A0V | M | 58 | PV | *JAK2 exon 12* | brother | PV |
| S146A10V | M | 40 | PV | *JAK2 V617F* | mother | PV |
| S15A10V | F | 56 | PV | *JAK2 V617F* | brother | PV |
| S17A10V | F | 52 | PV | *JAK2 V617F* | grandmother | PV |
| S19A6V | M | 52 | PV | *JAK2 V617F* | aunt | PV |
| S19A99V | M | 44 | PV | triple negative | father | PV |
| S1A10SM | F | 54 | PV | *JAK2 V617F* | daughter | PV |
| S1A10V | F | 53 | PV | *JAK2 V617F* | aunt | PV |
| S1A14V | F | 43 | PV | *JAK2 V617F* | mother | PV |
| S1A5V | F | 53 | PV | *JAK2 exon 12* | sister | PV |
| S23A8V | M | 13 | PV | *JAK2 V617F* | great uncle | PV |
| S25A4V | F | 35 | PV | *JAK2 exon 12* | father | PV |
| S48A3V | F | 56 | PV | *JAK2 V617F* | mother | PV |
| S58A11V | F | 63 | PV | *JAK2 V617F* | cousin | PV |
| S59A10V | F | 58 | PV | *JAK2 V617F* | mother | PV |
| S65A11V | F | 51 | PV | *JAK2 V617F* | father | PV |
| S71A15V | F | 39 | PV | *JAK2 V617F* | father | PV |
| S72A4V | M | 59 | PV | *JAK2 V617F* | sister | PV |
| S84A6V | M | 69 | PV | *JAK2 V617F* | brother | PV |
| S86A6V | F | 58 | PV | *JAK2 V617F* | son | PV |
| S107A8SM | F | 20 | ET | triple negative | father | AML |
| S162A14SM | F | 78 | ET | *JAK2 V617F* | daughter | AML |
| S182A7SM | M | 51 | ET | *JAK2 V617F* | father | AML |
| S189A15SM | M | 46 | ET | *CALR* | grandson | AML |
| S85A15SM | F | 38 | ET | *JAK2 V617F* | uncle | AML |
| S82A16SM | F | 29 | ET | *JAK2 V617F* | brother | B-ALL |
| S87A13SM | F | 24 | ET | *JAK2 V617F* | brother | B-ALL |
| S103A5SM | F | 32 | ET | triple negative | father | B-NHL |
| S129A9SM | M | 52 | ET | *CALR* | sister | B-NHL |
| S132A9SM | F | 19 | ET | *JAK2 V617F* | cousin | B-NHL |
| S15A13SM | F | 35 | ET | *JAK2 V617F* | mother | B-NHL |
| S165A7SM | M | 41 | ET | *JAK2 V617F* | father | B-NHL |
| S177A13SM | F | 53 | ET | *JAK2 V617F* | mother | B-NHL |
| S32A11SM | F | 68 | ET | triple negative | mother | B-NHL |
| S32A12SM | F | 53 | ET | *JAK2 V617F* | mother | B-NHL |
| S78A16SM | F | 55 | ET | *JAK2 V617F* | sister | B-NHL |
| S91A12SM | F | 55 | ET | *JAK2 V617F* | mother | B-NHL |
| S119A15SM | M | 52 | ET | *JAK2 V617F* | mother | CLL |
| S13A78SM | M | 38 | ET | not available | mother | CLL |
| S173A16SM | M | 40 | ET | *CALR* | cousin | CLL |
| S182A15SM | F | 53 | ET | *JAK2 V617F* | uncle | CLL |
| S53A1SM | F | 62 | ET | *JAK2 V617F* | cousin | CLL |
| S57A16SM | F | 64 | ET | *JAK2 V617F* | father | CLL |
| S88A16SM | M | 57 | ET | *JAK2 V617F* | aunt | CLL |
| S46A13SM | F | 47 | ET | *JAK2 V617F* | cousin | CML |
| S103A9SM | F | 67 | ET | triple negative | daughter | ET |
| S106A13SM | M | 85 | ET | *JAK2 V617F* | grand daughter | ET |
| S119A4SM | F | 65 | ET | *MPL* | sister | ET |
| S149A12SM | F | 59 | ET | *JAK2 V617F* | mother | ET |
| S16A9SM | F | 65 | ET | *JAK2 V617F* | sister | ET |
| S17A18SM | F | 46 | ET | *JAK2 V617F* | sister | ET |
| S20A10SM | F | 16 | ET | *CALR* | grandmother | ET |
| S25A9SM | F | 46 | ET | *JAK2 V617F* | cousin | ET |
| S37A3SM | F | 38 | ET | triple negative | mother | ET |
| S40A0SM | F | 38 | ET | *JAK2 V617F* | cousin | ET |
| S40A8SM | M | 63 | ET | triple negative | daughter | ET |
| S45A5V | M | 20 | ET | *JAK2 V617F* | father | ET |
| S53A8SM | F | 49 | ET | *JAK2 V617F* | father | ET |
| S60A8V | F | 43 | ET | *JAK2 V617F* | mother | ET |
| S75A2SM | M | 30 | ET | *JAK2 V617F* | mother | ET |
| S75A3SM | F | 16 | ET | *JAK2 V617F* | great uncle | ET |
| S76A2SM | F | 26 | ET | *JAK2 V617F* | father | ET |
| S76A99SM | F | 21 | ET | triple negative | cousin | ET |
| S93A10V | F | 61 | ET | *JAK2 V617F* | grandson | ET |
| S179A14SM | F | 51 | ET | *CALR* | daughter | HL |
| S20A15SM | F | 44 | ET | *MPL* | uncle | HL |
| S53A14SM | M | 48 | ET | *CALR* | brother | HL |
| S75A9SM | M | 35 | ET | *CALR* | sister | HL |
| S93A17SM | F | 38 | ET | *CALR* | aunt | HL |
| S1A12SM | F | 28 | ET | *JAK2 V617F* | cousin | Mastocytosis |
| S89A10SM | F | 52 | ET | *CALR* | father | MDS |
| S45A13SM | M | 71 | ET | *JAK2 V617F* | mother | MPN-U |
| S47A17SM | M | 44 | ET | *MPL* | uncle | PC neoplasm |
| S52A13SM | F | 32 | ET | triple negative | mother | PC neoplasm |
| S58A10SM | F | 62 | ET | *JAK2 V617F* | mother | PC neoplasm |
| S144A10SM | F | 33 | ET | *JAK2 V617F* | mother | PMF |
| S154A8SM | M | 50 | ET | *JAK2 V617F* | brother | PMF |
| S167A6SM | M | 34 | ET | *CALR* | uncle | PMF |
| S25A7SM | M | 51 | ET | *JAK2 V617F* | sister | PMF |
| S44A15SM | F | 22 | ET | *JAK2 V617F* | grandfather | PMF |
| S48A7SM | M | 55 | ET | *CALR* | sister | PMF |
| S60A7SM | F | 52 | ET | triple negative | brother | PMF |
| S78A11SM | F | 44 | ET | *JAK2 V617F* | sister | PMF |
| S91A6SM | M | 20 | ET | *JAK2 V617F* | great uncle | PMF |
| S93A11SM | M | 64 | ET | triple negative | sister | PMF |
| S107A8V | F | 63 | ET | *JAK2 V617F* | sister | PV |
| S119A10SM | M | 42 | ET | *JAK2 V617F* | father | PV |
| S121A14SM | M | 54 | ET | *JAK2 V617F* | mother | PV |
| S13A13SM | F | 39 | ET | *JAK2 V617F* | father | PV |
| S148A4SM | M | 31 | ET | *JAK2 V617F* | father | PV |
| S159A4SM | F | 63 | ET | *JAK2 V617F* | son | PV |
| S28A16SM | F | 66 | ET | *JAK2 V617F* | cousin | PV |
| S29A12SM | F | 46 | ET | *CALR* | mother | PV |
| S2A7SM | F | 37 | ET | *JAK2 V617F* | aunt | PV |
| S41A8SM | F | 34 | ET | *JAK2 V617F* | mother | PV |
| S80A10SM | F | 24 | ET | *JAK2 V617F* | mother | PV |
| S9A8V | M | 29 | ET | *JAK2 V617F* | father | PV |
| S34A85SM | F | 25 | ET | not available | sister | TCL |
| S54A6SM | F | 56 | PMF | *MPL* | father | AML |
| S94A1SM | F | 47 | PMF | *CALR* | brother | B-ALL |
| S18A12SM | M | 56 | PMF | *JAK2 V617F* | mother | B-NHL |
| S83A17SM | F | 50 | PMF | *JAK2 V617F* | uncle | B-NHL |
| S78A1SM | F | 37 | PMF | *CALR* | mother | CLL |
| S119A7SM | F | 76 | PMF | triple negative | daughter | ET |
| S14A6SM | M | 75 | PMF | *JAK2 V617F* | sister | ET |
| S17A99SM | M | 28 | PMF | *CALR* | mother | ET |
| S5A5SM | F | 57 | PMF | *JAK2 V617F* | mother | ET |
| S5A0SM | F | 21 | PMF | *JAK2 V617F* | father | HL |
| S157A10SM | M | 37 | PMF | *CALR* | father | MPN-U |
| S158A10SM | M | 33 | PMF | *JAK2 V617F* | father | MPN-U |
| S116A12SM | M | 38 | PMF | *CALR* | mother | PC neoplasm |
| S120A5SM | F | 43 | PMF | *CALR* | father | PC neoplasm |
| S94A16SM | F | 76 | PMF | *CALR* | brother | PC neoplasm |
| S120A8SM | M | 68 | PMF | triple negative | uncle | PMF |
| S166A5SM | M | 59 | PMF | *JAK2 V617F* | sister | PMF |
| S2A92SM | F | 23 | PMF | *JAK2 V617F* | father | PMF |
| S30A13SM | F | 49 | PMF | *CALR* | father | PMF |
| S48A9SM | M | 56 | PMF | *CALR* | brother | PMF |
| S55A5I | F | 66 | PMF | *JAK2 V617F* | brother | PMF |
| S58A15SM | F | 46 | PMF | *JAK2 V617F* | mother | PMF |
| S63A16SM | M | 66 | PMF | *JAK2 V617F* | grand daughter | PMF |
| S79A10SM | F | 63 | PMF | not available | mother | PMF |
| S14A2SM | F | 40 | PMF | *JAK2 V617F* | father | PV |
| S88A18SM | F | 61 | PMF | *CALR* | brother | PV |
| S55A9SM | F | 31 | MPN-U | *JAK2 V617F* | mother | B-NHL |
