# Supplementary material for: Haematological malignancies in relatives of patients affected with myeloproliferative neoplasms
Source: EJHaem. 2022 Mar 24;3(2):475–9. doi: 10.1002/jha2.425 (PMC9176120; doi:10.1002/jha2.425)
Supplement: Supplementary file 2 — Supporting Information [file JHA2-3-475-s002.docx]

Supplementary table 2 – Results of WES analysis showing the 65 putative germline variants with VAF values > 0.35 shared by both individuals of family #126

| Chr | Start | End | Ref | Alt | Func.refGene | Gene.refGene | MergeFunc.refGene | cytoBand | InterVar | AAChange.refGene |
| --- | --- | --- | --- | --- | --- | --- | --- | --- | --- | --- |
| 1 | 10166452 | 10166452 | C | T | exonic | UBE4B | nonsynonymous SNV | 1p36.22 | Uncertain significance | UBE4B:NM_001105562:exon7:c.C1007T:p.T336I |
| 1 | 185259915 | 185259915 | A | G | exonic | SWT1 | nonsynonymous SNV | 1q25.3 | Uncertain significance | SWT1:NM_001105518:exon19:c.A2683G:p.M895V, |
|  |  |  |  |  |  |  |  |  |  | SWT1:NM_017673:exon19:c.A2683G:p.M895V |
| 1 | 62582250 | 62582250 | G | A | exonic | PATJ | nonsynonymous SNV | 1p31.3 | Uncertain significance | PATJ:NM_001350145:exon36:c.G4702A:p.A1568T, |
|  |  |  |  |  |  |  |  |  |  | PATJ:NM_176877:exon36:c.G4702A:p.A1568T |
| 10 | 29769481 | 29769481 | T | C | exonic | SVIL | nonsynonymous SNV | 10p11.23 | Uncertain significance | SVIL:NM_003174:exon27:c.A4084G:p.M1362V, |
|  |  |  |  |  |  |  |  |  |  | SVIL:NM_001323600:exon28:c.A4180G:p.M1394V, |
|  |  |  |  |  |  |  |  |  |  | SVIL:NM_021738:exon29:c.A5362G:p.M1788V, |
|  |  |  |  |  |  |  |  |  |  | SVIL:NM_001323599:exon30:c.A4432G:p.M1478V |
| 10 | 46965887 | 46965887 | - | G | splicing | LOC102724488,SYT15 | NA | 10q11.22 | - | NA |
| 10 | 51768735 | 51768735 | A | G | exonic | AGAP6 | nonsynonymous SNV | 10q11.23 | Uncertain significance | AGAP6:NM_001077665:exon8:c.A850G:p.I284V, |
|  |  |  |  |  |  |  |  |  |  | AGAP6:NM_001365867:exon9:c.A247G:p.I83V |
| 11 | 132177687 | 132177687 | G | A | exonic | NTM | nonsynonymous SNV | 11q25 | Uncertain significance | NTM:NM_001144058:exon4:c.G631A:p.V211M, |
|  |  |  |  |  |  |  |  |  |  | NTM:NM_001144059:exon4:c.G631A:p.V211M, |
|  |  |  |  |  |  |  |  |  |  | NTM:NM_001352002:exon4:c.G631A:p.V211M, |
|  |  |  |  |  |  |  |  |  |  | NTM:NM_001352004:exon4:c.G631A:p.V211M, |
|  |  |  |  |  |  |  |  |  |  | NTM:NM_016522:exon4:c.G631A:p.V211M, |
|  |  |  |  |  |  |  |  |  |  | NTM:NM_001048209:exon5:c.G631A:p.V211M, |
|  |  |  |  |  |  |  |  |  |  | NTM:NM_001352001:exon5:c.G631A:p.V211M, |
|  |  |  |  |  |  |  |  |  |  | NTM:NM_001352003:exon5:c.G631A:p.V211M, |
|  |  |  |  |  |  |  |  |  |  | NTM:NM_001352005:exon5:c.G631A:p.V211M, |
|  |  |  |  |  |  |  |  |  |  | NTM:NM_001352008:exon5:c.G574A:p.V192M, |
|  |  |  |  |  |  |  |  |  |  | NTM:NM_001352009:exon5:c.G508A:p.V170M, |
|  |  |  |  |  |  |  |  |  |  | NTM:NM_001352006:exon6:c.G604A:p.V202M, |
|  |  |  |  |  |  |  |  |  |  | NTM:NM_001352007:exon6:c.G604A:p.V202M |
| 11 | 134104911 | 134104911 | C | T | exonic | VPS26B | nonsynonymous SNV | 11q25 | Uncertain significance | VPS26B:NM_052875:exon2:c.C344T:p.P115L |
| 11 | 3249269 | 3249269 | C | A | exonic | MRGPRE | nonsynonymous SNV | 11p15.4 | Uncertain significance | MRGPRE:NM_001039165:exon2:c.G761T:p.S254I |
| 11 | 67414358 | 67414358 | C | T | exonic | ACY3 | nonsynonymous SNV | 11q13.2 | Uncertain significance | ACY3:NM_080658:exon3:c.G157A:p.A53T |
| 11 | 7110770 | 7110770 | G | A | exonic | RBMXL2 | nonsynonymous SNV | 11p15.4 | Uncertain significance | RBMXL2:NM_014469:exon1:c.G419A:p.R140Q |
| 11 | 9879906 | 9879906 | C | G | exonic | SBF2 | nonsynonymous SNV | 11p15.4 | Uncertain significance | SBF2:NM_030962:exon18:c.G1967C:p.C656S |
| 12 | 120741450 | 120741450 | T | C | exonic | SIRT4 | nonsynonymous SNV | 12q24.31 | Uncertain significance | SIRT4:NM_012240:exon2:c.T86C:p.I29T |
| 12 | 122674765 | 122674765 | C | T | exonic | LRRC43 | stopgain | 12q24.31 | Uncertain significance | LRRC43:NM_001098519:exon5:c.C751T:p.R251X, |
|  |  |  |  |  |  |  |  |  |  | LRRC43:NM_152759:exon5:c.C196T:p.R66X |
| 12 | 132335619 | 132335619 | G | A | exonic | MMP17 | nonsynonymous SNV | 12q24.33 | Uncertain significance | MMP17:NM_016155:exon10:c.G1612A:p.A538T |
| 12 | 25705826 | 25705826 | T | C | exonic | LMNTD1 | nonsynonymous SNV | 12p12.1 | Uncertain significance | LMNTD1:NM_001145728:exon2:c.A68G:p.D23G, |
|  |  |  |  |  |  |  |  |  |  | LMNTD1:NM_001145729:exon2:c.A68G:p.D23G |
| 12 | 26731686 | 26731686 | T | G | exonic | ITPR2 | nonsynonymous SNV | 12p11.23 | Uncertain significance | ITPR2:NM_002223:exon34:c.A4590C:p.K1530N |
| 12 | 46757735 | 46757735 | C | T | exonic | SLC38A2 | nonsynonymous SNV | 12q13.11 | Uncertain significance | SLC38A2:NM_001307936:exon10:c.G625A:p.V209I, |
|  |  |  |  |  |  |  |  |  |  | SLC38A2:NM_018976:exon11:c.G925A:p.V309I |
| 12 | 48723180 | 48723180 | T | G | exonic | H1FNT | nonsynonymous SNV | 12q13.11 | Uncertain significance | H1FNT:NM_181788:exon1:c.T106G:p.S36A |
| 12 | 49691300 | 49691300 | A | G | exonic | PRPH | nonsynonymous SNV | 12q13.12 | Uncertain significance | PRPH:NM_006262:exon6:c.A1157G:p.K386R |
| 12 | 58112140 | 58112140 | G | A | exonic | OS9 | nonsynonymous SNV | 12q14.1 | Uncertain significance | OS9:NM_001261421:exon10:c.G1250A:p.R417Q, |
|  |  |  |  |  |  |  |  |  |  | OS9:NM_001261422:exon10:c.G1190A:p.R397Q, |
|  |  |  |  |  |  |  |  |  |  | OS9:NM_001261423:exon10:c.G1169A:p.R390Q, |
|  |  |  |  |  |  |  |  |  |  | OS9:NM_001017956:exon11:c.G1346A:p.R449Q, |
|  |  |  |  |  |  |  |  |  |  | OS9:NM_001017957:exon11:c.G1346A:p.R449Q, |
|  |  |  |  |  |  |  |  |  |  | OS9:NM_001017958:exon11:c.G1346A:p.R449Q, |
|  |  |  |  |  |  |  |  |  |  | OS9:NM_001261420:exon11:c.G1349A:p.R450Q, |
|  |  |  |  |  |  |  |  |  |  | OS9:NM_006812:exon11:c.G1346A:p.R449Q |
| 12 | 76844675 | 76844675 | T | C | exonic | OSBPL8 | nonsynonymous SNV | 12q21.2 | Uncertain significance | OSBPL8:NM_001003712:exon3:c.A47G:p.D16G, |
|  |  |  |  |  |  |  |  |  |  | OSBPL8:NM_001319653:exon4:c.A164G:p.D55G, |
|  |  |  |  |  |  |  |  |  |  | OSBPL8:NM_020841:exon4:c.A173G:p.D58G, |
|  |  |  |  |  |  |  |  |  |  | OSBPL8:NM_001319652:exon6:c.A47G:p.D16G |
| 13 | 51921325 | 51921325 | G | A | exonic | SERPINE3 | nonsynonymous SNV | 13q14.3 | Uncertain significance | SERPINE3:NM_001101320:exon3:c.G655A:p.V219I |
| 13 | 86369602 | 86369602 | G | A | exonic | SLITRK6 | stopgain | 13q31.1 | Uncertain significance | SLITRK6:NM_032229:exon2:c.C1042T:p.Q348X |
| 14 | 102675036 | 102675036 | T | C | exonic | WDR20 | nonsynonymous SNV | 14q32.31 | Uncertain significance | WDR20:NM_001242416:exon2:c.T346C:p.Y116H, |
|  |  |  |  |  |  |  |  |  |  | WDR20:NM_001353659:exon2:c.T382C:p.Y128H, |
|  |  |  |  |  |  |  |  |  |  | WDR20:NM_001353665:exon2:c.T10C:p.Y4H, |
|  |  |  |  |  |  |  |  |  |  | WDR20:NM_001353671:exon2:c.T10C:p.Y4H, |
|  |  |  |  |  |  |  |  |  |  | WDR20:NM_181308:exon2:c.T346C:p.Y116H, |
|  |  |  |  |  |  |  |  |  |  | WDR20:NM_001242418:exon3:c.T565C:p.Y189H, |
|  |  |  |  |  |  |  |  |  |  | WDR20:NM_001353656:exon3:c.T529C:p.Y177H, |
|  |  |  |  |  |  |  |  |  |  | WDR20:NM_001353657:exon3:c.T529C:p.Y177H, |
|  |  |  |  |  |  |  |  |  |  | WDR20:NM_001353662:exon3:c.T10C:p.Y4H, |
|  |  |  |  |  |  |  |  |  |  | WDR20:NM_001353664:exon3:c.T10C:p.Y4H, |
|  |  |  |  |  |  |  |  |  |  | WDR20:NM_001353668:exon3:c.T10C:p.Y4H, |
|  |  |  |  |  |  |  |  |  |  | WDR20:NM_001353674:exon3:c.T10C:p.Y4H, |
|  |  |  |  |  |  |  |  |  |  | WDR20:NM_144574:exon3:c.T529C:p.Y177H, |
|  |  |  |  |  |  |  |  |  |  | WDR20:NM_181291:exon3:c.T529C:p.Y177H, |
|  |  |  |  |  |  |  |  |  |  | WDR20:NM_001242417:exon4:c.T622C:p.Y208H, |
|  |  |  |  |  |  |  |  |  |  | WDR20:NM_001330228:exon4:c.T622C:p.Y208H, |
|  |  |  |  |  |  |  |  |  |  | WDR20:NM_001353658:exon4:c.T379C:p.Y127H, |
|  |  |  |  |  |  |  |  |  |  | WDR20:NM_001353660:exon4:c.T379C:p.Y127H, |
|  |  |  |  |  |  |  |  |  |  | WDR20:NM_001353661:exon4:c.T136C:p.Y46H, |
|  |  |  |  |  |  |  |  |  |  | WDR20:NM_001353663:exon4:c.T10C:p.Y4H, |
|  |  |  |  |  |  |  |  |  |  | WDR20:NM_001353666:exon4:c.T10C:p.Y4H, |
|  |  |  |  |  |  |  |  |  |  | WDR20:NM_001353669:exon4:c.T10C:p.Y4H, |
|  |  |  |  |  |  |  |  |  |  | WDR20:NM_001353670:exon4:c.T10C:p.Y4H, |
|  |  |  |  |  |  |  |  |  |  | WDR20:NM_001353672:exon4:c.T10C:p.Y4H, |
|  |  |  |  |  |  |  |  |  |  | WDR20:NM_001353667:exon5:c.T10C:p.Y4H, |
|  |  |  |  |  |  |  |  |  |  | WDR20:NM_001353673:exon5:c.T10C:p.Y4H |
| 14 | 105407762 | 105407762 | C | G | exonic | AHNAK2 | nonsynonymous SNV | 14q32.33 | Uncertain significance | AHNAK2:NM_001350929:exon7:c.G13726C:p.D4576H, |
|  |  |  |  |  |  |  |  |  |  | AHNAK2:NM_138420:exon7:c.G14026C:p.D4676H |
| 14 | 20014669 | 20014669 | C | T | exonic | POTEM | nonsynonymous SNV | 14q11.2 | Uncertain significance | POTEM:NM_001145442:exon3:c.G640A:p.V214I |
| 14 | 52186932 | 52186932 | C | T | exonic | FRMD6 | nonsynonymous SNV | 14q22.1 | Uncertain significance | FRMD6:NM_001267047:exon2:c.C110T:p.S37L, |
|  |  |  |  |  |  |  |  |  |  | FRMD6:NM_001267046:exon11:c.C1184T:p.S395L, |
|  |  |  |  |  |  |  |  |  |  | FRMD6:NM_152330:exon11:c.C1160T:p.S387L, |
|  |  |  |  |  |  |  |  |  |  | FRMD6:NM_001042481:exon12:c.C1160T:p.S387L |
| 14 | 59112926 | 59112926 | G | A | exonic | DACT1 | nonsynonymous SNV | 14q23.1 | Likely benign | DACT1:NM_001079520:exon4:c.G1474A:p.A492T, |
|  |  |  |  |  |  |  |  |  |  | DACT1:NM_016651:exon4:c.G1585A:p.A529T |
| 14 | 59988376 | 59988376 | T | G | exonic | CCDC175 | nonsynonymous SNV | 14q23.1 | Uncertain significance | CCDC175:NM_001164399:exon17:c.A2014C:p.N672H |
| 15 | 34145853 | 34145853 | C | T | exonic | RYR3 | nonsynonymous SNV | 15q14 | Uncertain significance | RYR3:NM_001243996:exon95:c.C13754T:p.T4585I, |
|  |  |  |  |  |  |  |  |  |  | RYR3:NM_001036:exon96:c.C13769T:p.T4590I |
| 15 | 74574186 | 74574186 | C | G | exonic | CCDC33 | nonsynonymous SNV | 15q24.1 | Uncertain significance | CCDC33:NM_025055:exon10:c.C1091G:p.S364C |
| 16 | 1250294 | 1250294 | C | T | exonic | CACNA1H | nonsynonymous SNV | 16p13.3 | Uncertain significance | CACNA1H:NM_001005407:exon7:c.C842T:p.T281M, |
|  |  |  |  |  |  |  |  |  |  | CACNA1H:NM_021098:exon7:c.C842T:p.T281M |
| 16 | 2230851 | 2230851 | C | T | exonic | CASKIN1 | nonsynonymous SNV | 16p13.3 | Uncertain significance | CASKIN1:NM_020764:exon18:c.G2518A:p.V840M |
| 16 | 4871577 | 4871577 | T | C | exonic | GLYR1 | nonsynonymous SNV | 16p13.3 | Uncertain significance | GLYR1:NM_001324096:exon7:c.A460G:p.I154V, |
|  |  |  |  |  |  |  |  |  |  | GLYR1:NM_001324097:exon7:c.A460G:p.I154V, |
|  |  |  |  |  |  |  |  |  |  | GLYR1:NM_001308096:exon8:c.A703G:p.I235V, |
|  |  |  |  |  |  |  |  |  |  | GLYR1:NM_032569:exon8:c.A703G:p.I235V |
| 16 | 88503462 | 88503462 | G | A | exonic | ZNF469 | nonsynonymous SNV | 16q24.2 | Uncertain significance | ZNF469:NM_001367624:exon1:c.G9584A:p.R3195H |
| 19 | 33355167 | 33355167 | C | T | exonic | SLC7A9 | nonsynonymous SNV | 19q13.11 | Uncertain significance | SLC7A9:NM_001126335:exon4:c.G313A:p.G105R, |
|  |  |  |  |  |  |  |  |  |  | SLC7A9:NM_001243036:exon4:c.G313A:p.G105R, |
|  |  |  |  |  |  |  |  |  |  | SLC7A9:NM_014270:exon4:c.G313A:p.G105R |
| 19 | 48699237 | 48699237 | G | A | exonic | ZSWIM9 | nonsynonymous SNV | 19q13.33 | Uncertain significance | ZSWIM9:NM_199341:exon4:c.G1916A:p.G639E |
| 19 | 50519407 | 50519407 | A | G | exonic | VRK3 | nonsynonymous SNV | 19q13.33 | Uncertain significance | VRK3:NM_001025778:exon3:c.T13C:p.C5R, |
|  |  |  |  |  |  |  |  |  |  | VRK3:NM_001308420:exon3:c.T13C:p.C5R, |
|  |  |  |  |  |  |  |  |  |  | VRK3:NM_016440:exon3:c.T13C:p.C5R |
| 19 | 51483678 | 51483678 | G | C | exonic | KLK7 | nonsynonymous SNV | 19q13.41 | Uncertain significance | KLK7:NM_001207053:exon3:c.C71G:p.S24W, |
|  |  |  |  |  |  |  |  |  |  | KLK7:NM_001243126:exon3:c.C266G:p.S89W, |
|  |  |  |  |  |  |  |  |  |  | KLK7:NM_005046:exon4:c.C287G:p.S96W, |
|  |  |  |  |  |  |  |  |  |  | KLK7:NM_139277:exon4:c.C287G:p.S96W |
| 19 | 51582098 | 51582098 | G | A | exonic | KLK14 | nonsynonymous SNV | 19q13.41 | Uncertain significance | KLK14:NM_001369775:exon5:c.C577T:p.P193S, |
|  |  |  |  |  |  |  |  |  |  | KLK14:NM_001311182:exon6:c.C577T:p.P193S, |
|  |  |  |  |  |  |  |  |  |  | KLK14:NM_022046:exon6:c.C577T:p.P193S |
| 19 | 54933577 | 54933577 | G | A | exonic | TTYH1 | nonsynonymous SNV | 19q13.42 | Uncertain significance | TTYH1:NM_001005367:exon4:c.G631A:p.E211K, |
|  |  |  |  |  |  |  |  |  |  | TTYH1:NM_001201461:exon4:c.G631A:p.E211K, |
|  |  |  |  |  |  |  |  |  |  | TTYH1:NM_020659:exon4:c.G631A:p.E211K |
| 2 | 108477717 | 108477717 | G | A | exonic | RGPD4 | nonsynonymous SNV | 2q12.3 | Uncertain significance | RGPD4:NM_182588:exon14:c.G1988A:p.G663E |
| 2 | 219495439 | 219495439 | G | T | exonic | PLCD4 | nonsynonymous SNV | 2q35 | Uncertain significance | PLCD4:NM_032726:exon9:c.G1186T:p.A396S |
| 2 | 219507737 | 219507737 | G | A | exonic | ZNF142 | nonsynonymous SNV | 2q35 | Uncertain significance | ZNF142:NM_001366291:exon7:c.C3502T:p.R1168W, |
|  |  |  |  |  |  |  |  |  |  | ZNF142:NM_001105537:exon8:c.C3502T:p.R1168W, |
|  |  |  |  |  |  |  |  |  |  | ZNF142:NM_001366290:exon8:c.C4102T:p.R1368W, |
|  |  |  |  |  |  |  |  |  |  | ZNF142:NM_001366287:exon9:c.C3013T:p.R1005W, |
|  |  |  |  |  |  |  |  |  |  | ZNF142:NM_001366288:exon9:c.C3013T:p.R1005W, |
|  |  |  |  |  |  |  |  |  |  | ZNF142:NM_001366289:exon9:c.C3013T:p.R1005W |
| 2 | 227973309 | 227973309 | T | G | exonic | COL4A4 | nonsynonymous SNV | 2q36.3 | Uncertain significance | COL4A4:NM_000092:exon12:c.A723C:p.Q241H |
| 2 | 233398720 | 233398720 | G | A | exonic | CHRND | nonsynonymous SNV | 2q37.1 | Uncertain significance | CHRND:NM_001311195:exon8:c.G545A:p.R182Q, |
|  |  |  |  |  |  |  |  |  |  | CHRND:NM_001256657:exon9:c.G1082A:p.R361Q, |
|  |  |  |  |  |  |  |  |  |  | CHRND:NM_000751:exon10:c.G1127A:p.R376Q, |
|  |  |  |  |  |  |  |  |  |  | CHRND:NM_001311196:exon10:c.G824A:p.R275Q |
| 2 | 74074539 | 74074539 | G | A | exonic | STAMBP | nonsynonymous SNV | 2p13.1 | Uncertain significance | STAMBP:NM_001353968:exon5:c.G401A:p.R134Q, |
|  |  |  |  |  |  |  |  |  |  | STAMBP:NM_001353969:exon5:c.G401A:p.R134Q, |
|  |  |  |  |  |  |  |  |  |  | STAMBP:NM_201647:exon5:c.G401A:p.R134Q, |
|  |  |  |  |  |  |  |  |  |  | STAMBP:NM_213622:exon5:c.G401A:p.R134Q, |
|  |  |  |  |  |  |  |  |  |  | STAMBP:NM_001353967:exon6:c.G401A:p.R134Q, |
|  |  |  |  |  |  |  |  |  |  | STAMBP:NM_001353970:exon6:c.G401A:p.R134Q, |
|  |  |  |  |  |  |  |  |  |  | STAMBP:NM_006463:exon6:c.G401A:p.R134Q |
| 2 | 85820108 | 85820110 | AGA | - | exonic | VAMP5 | nonframeshift deletion | 2p11.2 | - | VAMP5:NM_006634:exon3:c.179_181del:p.60_61del |
| 2 | 85981410 | 85981410 | C | A | exonic | ATOH8 | nonsynonymous SNV | 2p11.2 | Uncertain significance | ATOH8:NM_032827:exon1:c.C98A:p.P33Q |
| 2 | 95542378 | 95542378 | G | A | exonic | TEKT4 | nonsynonymous SNV | 2q11.1 | Uncertain significance | TEKT4:NM_001286559:exon6:c.G626A:p.R209H, |
|  |  |  |  |  |  |  |  |  |  | TEKT4:NM_144705:exon6:c.G1172A:p.R391H |
| 2 | 96789896 | 96789896 | G | T | exonic | ASTL | nonsynonymous SNV | 2q11.1 | Uncertain significance | ASTL:NM_001002036:exon9:c.C989A:p.A330E |
| 2 | 99006149 | 99006149 | G | A | exonic | CNGA3 | nonsynonymous SNV | 2q11.2 | Uncertain significance | CNGA3:NM_001079878:exon5:c.G424A:p.V142M, |
|  |  |  |  |  |  |  |  |  |  | CNGA3:NM_001298:exon6:c.G478A:p.V160M |
| 20 | 40040800 | 40040800 | T | C | exonic | CHD6 | nonsynonymous SNV | 20q12 | Uncertain significance | CHD6:NM_032221:exon36:c.A7235G:p.K2412R |
| 4 | 2130962 | 2130962 | G | A | exonic | POLN | nonsynonymous SNV | 4p16.3 | Uncertain significance | POLN:NM_181808:exon18:c.C1811T:p.T604M |
| 4 | 3534129 | 3534129 | C | T | exonic | LRPAP1 | nonsynonymous SNV | 4p16.3 | Likely benign | LRPAP1:NM_002337:exon1:c.G11A:p.R4Q |
| 4 | 8442652 | 8442652 | A | G | exonic | TRMT44 | nonsynonymous SNV | 4p16.1 | Uncertain significance | TRMT44:NM_152544:exon1:c.A103G:p.N35D |
| 5 | 168114030 | 168114030 | C | T | exonic | SLIT3 | nonsynonymous SNV | 5q34 | Uncertain significance | SLIT3:NM_001271946:exon30:c.G3289A:p.V1097M, |
|  |  |  |  |  |  |  |  |  |  | SLIT3:NM_003062:exon30:c.G3268A:p.V1090M |
| 5 | 23527565 | 23527565 | A | C | exonic | PRDM9 | nonsynonymous SNV | 5p14.2 | Likely benign | PRDM9:NM_001310214:exon11:c.A2368C:p.N790H, |
|  |  |  |  |  |  |  |  |  |  | PRDM9:NM_020227:exon11:c.A2368C:p.N790H |
| 5 | 5235264 | 5235264 | G | A | exonic | ADAMTS16 | nonsynonymous SNV | 5p15.32 | Uncertain significance | ADAMTS16:NM_139056:exon13:c.G1988A:p.R663Q |
| 5 | 65892767 | 65892767 | - | GCC | exonic | MAST4 | nonframeshift insertion | 5q12.3 | - | MAST4:NM_001164664:exon1:c.284_285insGCC:p.L95delinsLP, |
|  |  |  |  |  |  |  |  |  |  | MAST4:NM_001290228:exon1:c.284_285insGCC:p.L95delinsLP, |
|  |  |  |  |  |  |  |  |  |  | MAST4:NM_198828:exon1:c.284_285insGCC:p.L95delinsLP |
| 7 | 103270596 | 103270596 | A | C | exonic | RELN | nonsynonymous SNV | 7q22.1 | Uncertain significance | RELN:NM_005045:exon20:c.T2493G:p.D831E, |
|  |  |  |  |  |  |  |  |  |  | RELN:NM_173054:exon20:c.T2493G:p.D831E |
| 7 | 5385208 | 5385208 | G | A | exonic | TNRC18 | nonsynonymous SNV | 7p22.1 | Uncertain significance | TNRC18:NM_001080495:exon18:c.C5704T:p.R1902W |
| 8 | 62588645 | 62588645 | G | A | exonic | ASPH | nonsynonymous SNV | 8q12.3 | Uncertain significance | ASPH:NM_001164756:exon5:c.C601T:p.H201Y |
| X | 96139850 | 96139850 | C | A | exonic | RPA4 | nonsynonymous SNV | Xq21.33 | Uncertain significance | RPA4:NM_013347:exon1:c.C541A:p.P181T |
