# Supplementary material for: Haematological malignancies in relatives of patients affected with myeloproliferative neoplasms
Source: EJHaem. 2022 Mar 24;3(2):475–9. doi: 10.1002/jha2.425 (PMC9176120; doi:10.1002/jha2.425)
Supplement: Supplementary file 3 — Supporting Information [file JHA2-3-475-s004.docx]

Supplementary table 3 - Results of WES analysis showing the 147 putative germline variants with VAF values > 0.35 shared by both individuals of family #127

| Chr | Start | End | Ref | Alt | Func.refGene | Gene.refGene | Merge_Func | cytoBand | InterVar_automated | AAChange.refGene |
| --- | --- | --- | --- | --- | --- | --- | --- | --- | --- | --- |
| 1 | 108742688 | 108742688 | C | A | exonic | SLC25A24 | stopgain | 1p13.3 | Uncertain significance | SLC25A24:NM_013386:exon1:c.G73T:p.E25X |
| 1 | 114454742 | 114454742 | A | T | exonic | DCLRE1B | nonsynonymous SNV | 1p13.2 | Uncertain significance | DCLRE1B:NM_001319946:exon3:c.A1150T:p.N384Y, DCLRE1B:NM_001319947:exon4:c.A1150T:p.N384Y, DCLRE1B:NM_022836:exon4:c.A1528T:p.N510Y |
| 1 | 120295237 | 120295237 | C | T | exonic | HMGCS2 | nonsynonymous SNV | 1p12 | Uncertain significance | HMGCS2:NM_001166107:exon7:c.G1229A:p.R410Q, HMGCS2:NM_005518:exon8:c.G1355A:p.R452Q |
| 1 | 147090775 | 147090775 | C | T | exonic | BCL9 | nonsynonymous SNV | 1q21.2 | Uncertain significance | BCL9:NM_004326:exon8:c.C814T:p.R272C |
| 1 | 150960594 | 150960594 | G | A | exonic | ANXA9 | nonsynonymous SNV | 1q21.3 | Uncertain significance | ANXA9:NM_003568:exon11:c.G725A:p.G242E |
| 1 | 151584734 | 151584734 | - | GGC | exonic | SNX27 | nonframeshift insertion | 1q21.3 | - | SNX27:NM_001330723:exon1:c.57_58insGGC:p.G19delinsGG, SNX27:NM_030918:exon1:c.57_58insGGC:p.G19delinsGG |
| 1 | 175129955 | 175129955 | G | T | exonic | KIAA0040 | nonsynonymous SNV | 1q25.1 | Uncertain significance | KIAA0040:NM_001162895:exon3:c.C195A:p.N65K, KIAA0040:NM_001319230:exon3:c.C195A:p.N65K, KIAA0040:NM_001162894:exon4:c.C195A:p.N65K, KIAA0040:NM_014656:exon4:c.C195A:p.N65K, KIAA0040:NM_001162893:exon5:c.C195A:p.N65K, KIAA0040:NM_001319231:exon5:c.C195A:p.N65K |
| 1 | 176833427 | 176833427 | T | C | exonic | ASTN1 | nonsynonymous SNV | 1q25.2 | Uncertain significance | ASTN1:NM_001364856:exon23:c.A3902G:p.E1301G, ASTN1:NM_004319:exon23:c.A3878G:p.E1293G |
| 1 | 222827636 | 222827636 | C | T | exonic | MIA3 | nonsynonymous SNV | 1q41 | Uncertain significance | MIA3:NM_001300867:exon11:c.C1007T:p.S336F, MIA3:NM_001324065:exon11:c.C1007T:p.S336F, MIA3:NM_001324063:exon14:c.C4196T:p.S1399F, MIA3:NM_001324062:exon16:c.C4373T:p.S1458F, MIA3:NM_198551:exon16:c.C4373T:p.S1458F, MIA3:NM_001324064:exon17:c.C3881T:p.S1294F |
| 1 | 245766077 | 245766077 | G | A | exonic | KIF26B | nonsynonymous SNV | 1q44 | Uncertain significance | KIF26B:NM_018012:exon6:c.G1549A:p.A517T |
| 1 | 247492555 | 247492555 | C | T | exonic | ZNF496 | nonsynonymous SNV | 1q44 | Uncertain significance | ZNF496:NM_001329733:exon4:c.G326A:p.S109N, ZNF496:NM_032752:exon4:c.G326A:p.S109N |
| 1 | 247751843 | 247751843 | C | T | exonic | OR2G2 | nonsynonymous SNV | 1q44 | Uncertain significance | OR2G2:NM_001001915:exon1:c.C182T:p.P61L |
| 1 | 32479816 | 32479816 | C | T | exonic | KHDRBS1 | nonsynonymous SNV | 1p35.1 | Uncertain significance | KHDRBS1:NM_001271878:exon1:c.C220T:p.P74S, KHDRBS1:NM_006559:exon1:c.C220T:p.P74S |
| 1 | 33945011 | 33945011 | G | A | exonic | ZSCAN20 | nonsynonymous SNV | 1p35.1 | Likely benign | ZSCAN20:NM_145238:exon2:c.G122A:p.R41H |
| 1 | 33958853 | 33958853 | C | T | exonic | ZSCAN20 | nonsynonymous SNV | 1p35.1 | Uncertain significance | ZSCAN20:NM_145238:exon7:c.C1511T:p.S504L |
| 1 | 77629532 | 77629532 | G | A | exonic | PIGK | nonsynonymous SNV | 1p31.1 | Uncertain significance | PIGK:NM_005482:exon6:c.C583T:p.R195C |
| 10 | 102249520 | 102249520 | T | C | splicing | SEC31B | splicing | 10q24.31 | - | NA |
| 10 | 118616073 | 118616073 | C | T | exonic | ENO4 | nonsynonymous SNV | 10q25.3 | Uncertain significance | ENO4:NM_001242699:exon3:c.C365T:p.A122V |
| 10 | 16989271 | 16989271 | C | G | exonic | CUBN | nonsynonymous SNV | 10p13 | Likely benign | CUBN:NM_001081:exon36:c.G5305C:p.V1769L |
| 10 | 61842379 | 61842379 | A | C | exonic | ANK3 | nonsynonymous SNV | 10q21.2 | Uncertain significance | ANK3:NM_001149:exon12:c.T1719G:p.H573Q, ANK3:NM_001320874:exon34:c.T4317G:p.H1439Q, ANK3:NM_020987:exon34:c.T4317G:p.H1439Q, ANK3:NM_001204403:exon35:c.T4299G:p.H1433Q, ANK3:NM_001204404:exon35:c.T4320G:p.H1440Q |
| 10 | 75434973 | 75434973 | T | C | exonic | AGAP5 | nonsynonymous SNV | 10q22.2 | Uncertain significance | AGAP5:NM_001144000:exon8:c.A1445G:p.Y482C |
| 11 | 93844214 | 93844214 | C | T | exonic | HEPHL1 | nonsynonymous SNV | 11q21 | Uncertain significance | HEPHL1:NM_001098672:exon18:c.C3191T:p.T1064M |
| 12 | 124371801 | 124371801 | C | T | exonic | DNAH10 | nonsynonymous SNV | 12q24.31 | Uncertain significance | DNAH10:NM_207437:exon51:c.C8582T:p.T2861M |
| 12 | 12940234 | 12940234 | G | T | exonic | APOLD1 | nonsynonymous SNV | 12p13.1 | Uncertain significance | APOLD1:NM_001130415:exon2:c.G488T:p.R163L, APOLD1:NM_030817:exon2:c.G395T:p.R132L |
| 12 | 132511987 | 132511987 | G | A | exonic | EP400 | nonsynonymous SNV | 12q24.33 | Uncertain significance | EP400:NM_015409:exon26:c.G5020A:p.V1674I |
| 12 | 6976650 | 6976650 | C | T | exonic | TPI1 | nonsynonymous SNV | 12p13.31 | Likely benign | TPI1:NM_001159287:exon1:c.C31T:p.H11Y |
| 12 | 69983450 | 69983450 | A | G | exonic | CCT2 | nonsynonymous SNV | 12q15 | Uncertain significance | CCT2:NM_001198842:exon7:c.A491G:p.D164G, CCT2:NM_006431:exon7:c.A632G:p.D211G |
| 13 | 38154813 | 38154813 | A | G | exonic | POSTN | nonsynonymous SNV | 13q13.3 | Uncertain significance | POSTN:NM_001135934:exon11:c.T1414C:p.C472R, POSTN:NM_001135935:exon11:c.T1414C:p.C472R, POSTN:NM_001135936:exon11:c.T1414C:p.C472R, POSTN:NM_001286665:exon11:c.T1414C:p.C472R, POSTN:NM_001286666:exon11:c.T1414C:p.C472R, POSTN:NM_001286667:exon11:c.T1414C:p.C472R, POSTN:NM_001330517:exon11:c.T1414C:p.C472R, POSTN:NM_006475:exon11:c.T1414C:p.C472R |
| 13 | 64406942 | 64406942 | G | C | exonic | LOC647264 | nonsynonymous SNV | 13q21.31 | - | LOC647264:NM_001370368:exon1:c.C185G:p.S62C |
| 14 | 105060626 | 105060626 | C | T | exonic | TMEM179 | stopgain | 14q32.33 | Uncertain significance | TMEM179:NM_001286390:exon4:c.G629A:p.W210X |
| 14 | 105417725 | 105417725 | C | G | exonic | AHNAK2 | nonsynonymous SNV | 14q32.33 | Uncertain significance | AHNAK2:NM_001350929:exon7:c.G3763C:p.A1255P, AHNAK2:NM_138420:exon7:c.G4063C:p.A1355P |
| 14 | 21792933 | 21792933 | C | T | exonic | RPGRIP1 | nonsynonymous SNV | 14q11.2 | Uncertain significance | RPGRIP1:NM_020366:exon14:c.C1919T:p.A640V |
| 14 | 24114454 | 24114454 | C | T | exonic | DHRS2 | nonsynonymous SNV | 14q11.2 | Uncertain significance | DHRS2:NM_005794:exon9:c.C835T:p.R279W |
| 14 | 24551745 | 24551745 | C | T | exonic | NRL | nonsynonymous SNV | 14q11.2 | Uncertain significance | NRL:NM_001354768:exon2:c.G313A:p.V105I, NRL:NM_001354769:exon3:c.G313A:p.V105I, NRL:NM_006177:exon3:c.G313A:p.V105I |
| 14 | 24600933 | 24600933 | G | A | exonic | FITM1 | nonsynonymous SNV | 14q12 | Uncertain significance | FITM1:NM_203402:exon1:c.G161A:p.R54H |
| 14 | 24633293 | 24633293 | C | T | exonic | IRF9 | nonsynonymous SNV | 14q12 | Uncertain significance | IRF9:NM_006084:exon6:c.C599T:p.P200L |
| 14 | 52493997 | 52493997 | G | A | exonic | NID2 | nonsynonymous SNV | 14q22.1 | Uncertain significance | NID2:NM_007361:exon12:c.C2596T:p.R866W |
| 14 | 52496343 | 52496343 | G | A | exonic | NID2 | nonsynonymous SNV | 14q22.1 | Uncertain significance | NID2:NM_007361:exon10:c.C2323T:p.R775W |
| 14 | 75230410 | 75230410 | A | G | exonic | YLPM1 | nonsynonymous SNV | 14q24.3 | Uncertain significance | YLPM1:NM_019589:exon1:c.A218G:p.Q73R |
| 14 | 88651919 | 88651919 | C | T | exonic | KCNK10 | nonsynonymous SNV | 14q31.3 | Uncertain significance | KCNK10:NM_021161:exon7:c.G1577A:p.R526Q, KCNK10:NM_138317:exon7:c.G1592A:p.R531Q, KCNK10:NM_138318:exon7:c.G1592A:p.R531Q |
| 15 | 30437693 | 30437693 | C | G | exonic | GOLGA8T | nonsynonymous SNV | 15q13.2 | - | GOLGA8T:NM_001355469:exon19:c.C1819G:p.Q607E |
| 15 | 49320694 | 49320694 | G | A | exonic | SECISBP2L | nonsynonymous SNV | 15q21.1 | Uncertain significance | SECISBP2L:NM_001193489:exon5:c.C850T:p.P284S, SECISBP2L:NM_014701:exon5:c.C850T:p.P284S |
| 15 | 65110155 | 65110155 | T | G | exonic | PIF1 | nonsynonymous SNV | 15q22.31 | Uncertain significance | PIF1:NM_001286496:exon11:c.A1664C:p.H555P, PIF1:NM_001286497:exon11:c.A1664C:p.H555P, PIF1:NM_001286499:exon11:c.A1505C:p.H502P, PIF1:NM_025049:exon11:c.A1664C:p.H555P |
| 15 | 67477118 | 67477118 | A | G | exonic | SMAD3 | nonsynonymous SNV | 15q22.33 | Uncertain significance | SMAD3:NM_001145104:exon5:c.A340G:p.S114G, SMAD3:NM_001145102:exon7:c.A610G:p.S204G, SMAD3:NM_001145103:exon7:c.A793G:p.S265G, SMAD3:NM_005902:exon7:c.A925G:p.S309G |
| 15 | 77233976 | 77233976 | A | G | splicing | RCN2 | splicing | 15q24.3 | - | NA |
| 16 | 47005323 | 47005323 | C | T | exonic | DNAJA2 | nonsynonymous SNV | 16q12.1 | Uncertain significance | DNAJA2:NM_005880:exon3:c.G300A:p.M100I |
| 16 | 57597818 | 57597818 | G | A | exonic | ADGRG5 | nonsynonymous SNV | 16q21 | Uncertain significance | ADGRG5:NM_001304376:exon5:c.G356A:p.R119Q, ADGRG5:NM_001318481:exon5:c.G356A:p.R119Q, ADGRG5:NM_153837:exon5:c.G356A:p.R119Q |
| 16 | 57973384 | 57973384 | G | A | exonic | CNGB1 | nonsynonymous SNV | 16q21 | Uncertain significance | CNGB1:NM_001286130:exon16:c.C1304T:p.A435V, CNGB1:NM_001297:exon16:c.C1322T:p.A441V |
| 16 | 69718836 | 69718836 | T | G | exonic | NFAT5 | nonsynonymous SNV | 16q22.1 | Uncertain significance | NFAT5:NM_001367709:exon10:c.T1062G:p.S354R, NFAT5:NM_006599:exon10:c.T1683G:p.S561R, NFAT5:NM_001113178:exon11:c.T1734G:p.S578R, NFAT5:NM_138713:exon11:c.T1737G:p.S579R, NFAT5:NM_173214:exon11:c.T1455G:p.S485R, NFAT5:NM_173215:exon11:c.T1455G:p.S485R, NFAT5:NM_138714:exon12:c.T1455G:p.S485R |
| 17 | 1174332 | 1174332 | A | G | exonic | BHLHA9 | nonsynonymous SNV | 17p13.3 | Uncertain significance | BHLHA9:NM_001164405:exon1:c.A475G:p.S159G |
| 17 | 26856201 | 26856201 | T | G | exonic | FOXN1 | nonsynonymous SNV | 17q11.2 | Uncertain significance | FOXN1:NM_003593:exon4:c.T789G:p.D263E, FOXN1:NM_001369369:exon5:c.T789G:p.D263E |
| 17 | 3144623 | 3144623 | T | G | exonic | OR1D4 | unknown | 17p13.3 | - | UNKNOWN |
| 17 | 3300929 | 3300929 | T | C | exonic | OR1E1 | nonsynonymous SNV | 17p13.2 | Uncertain significance | OR1E1:NM_003553:exon1:c.A776G:p.Y259C |
| 17 | 42743946 | 42743946 | C | T | exonic | MEIOC | nonsynonymous SNV | 17q21.31 | Uncertain significance | MEIOC:NM_001145080:exon5:c.C667T:p.L223F |
| 17 | 45897102 | 45897102 | C | T | exonic | OSBPL7 | nonsynonymous SNV | 17q21.32 | Uncertain significance | OSBPL7:NM_145798:exon4:c.G248A:p.R83Q |
| 17 | 5064882 | 5064882 | A | G | exonic | USP6 | nonsynonymous SNV | 17p13.2 | Uncertain significance | USP6:NM_004505:exon24:c.A2888G:p.N963S, USP6:NM_001304284:exon32:c.A2888G:p.N963S |
| 17 | 56599320 | 56599320 | G | T | exonic | SEPTIN4 | nonsynonymous SNV | 17q22 | Uncertain significance | SEPTIN4:NM_080415:exon6:c.C748A:p.P250T |
| 17 | 59483099 | 59483099 | G | C | exonic | TBX2 | nonsynonymous SNV | 17q23.2 | Uncertain significance | TBX2:NM_005994:exon6:c.G1588C:p.A530P |
| 17 | 59968900 | 59968900 | G | A | exonic | INTS2 | nonsynonymous SNV | 17q23.2 | Uncertain significance | INTS2:NM_001330417:exon14:c.C1849T:p.L617F, INTS2:NM_001351695:exon14:c.C1849T:p.L617F, INTS2:NM_020748:exon14:c.C1873T:p.L625F |
| 17 | 61497373 | 61497373 | G | A | exonic | TANC2 | nonsynonymous SNV | 17q23.3 | Uncertain significance | TANC2:NM_025185:exon26:c.G4030A:p.A1344T |
| 17 | 63533913 | 63533913 | C | T | exonic | AXIN2 | nonsynonymous SNV | 17q24.1 | Uncertain significance | AXIN2:NM_001363813:exon6:c.G1241A:p.R414Q, AXIN2:NM_004655:exon6:c.G1241A:p.R414Q |
| 17 | 64219816 | 64219816 | G | A | exonic | APOH | nonsynonymous SNV | 17q24.2 | Uncertain significance | APOH:NM_000042:exon4:c.C415T:p.P139S |
| 17 | 7128292 | 7128292 | G | A | exonic | ACADVL | nonsynonymous SNV | 17p13.1 | Uncertain significance | ACADVL:NM_001033859:exon19:c.G1778A:p.R593Q, ACADVL:NM_001270448:exon19:c.G1616A:p.R539Q, ACADVL:NM_000018:exon20:c.G1844A:p.R615Q, ACADVL:NM_001270447:exon21:c.G1913A:p.R638Q |
| 17 | 78069140 | 78069140 | G | A | exonic | CCDC40 | nonsynonymous SNV | 17q25.3 | Likely benign | CCDC40:NM_017950:exon18:c.G2911A:p.V971I |
| 18 | 12836807 | 12836807 | T | C | exonic | PTPN2 | nonsynonymous SNV | 18p11.21 | Uncertain significance | PTPN2:NM_001308287:exon2:c.A157G:p.S53G, PTPN2:NM_001207013:exon3:c.A244G:p.S82G, PTPN2:NM_002828:exon3:c.A244G:p.S82G, PTPN2:NM_080422:exon3:c.A244G:p.S82G, PTPN2:NM_080423:exon3:c.A244G:p.S82G |
| 18 | 21519250 | 21519250 | C | A | exonic | LAMA3 | nonsynonymous SNV | 18q11.2 | Likely benign | LAMA3:NM_001127718:exon30:c.C3931A:p.P1311T, LAMA3:NM_000227:exon31:c.C4099A:p.P1367T, LAMA3:NM_001127717:exon67:c.C8758A:p.P2920T, LAMA3:NM_198129:exon68:c.C8926A:p.P2976T |
| 18 | 33706517 | 33706517 | C | A | exonic | SLC39A6 | nonsynonymous SNV | 18q12.2 | Uncertain significance | SLC39A6:NM_012319:exon2:c.G454T:p.D152Y |
| 18 | 34232736 | 34232736 | G | A | exonic | FHOD3 | nonsynonymous SNV | 18q12.2 | Uncertain significance | FHOD3:NM_001281740:exon12:c.G1490A:p.R497Q |
| 19 | 12541268 | 12541268 | G | A | exonic | ZNF443 | nonsynonymous SNV | 19p13.2 | Uncertain significance | ZNF443:NM_005815:exon4:c.C1718T:p.T573I |
| 19 | 2934625 | 2934625 | C | T | exonic | ZNF77 | nonsynonymous SNV | 19p13.3 | Uncertain significance | ZNF77:NM_021217:exon4:c.G500A:p.C167Y |
| 19 | 49147796 | 49147796 | G | A | exonic | CA11 | nonsynonymous SNV | 19q13.33 | Uncertain significance | CA11:NM_001217:exon3:c.C173T:p.A58V |
| 19 | 49812952 | 49812952 | G | C | exonic | SLC6A16 | nonsynonymous SNV | 19q13.33 | Likely benign | SLC6A16:NM_014037:exon5:c.C832G:p.L278V |
| 19 | 56658321 | 56658321 | T | C | exonic | ZNF444 | nonsynonymous SNV | 19q13.43 | Uncertain significance | ZNF444:NM_001253792:exon3:c.T41C:p.L14P, ZNF444:NM_018337:exon3:c.T41C:p.L14P |
| 19 | 578014 | 578014 | C | T | exonic | BSG | nonsynonymous SNV | 19p13.3 | Uncertain significance | BSG:NM_001728:exon2:c.C308T:p.T103M |
| 19 | 5791071 | 5791071 | C | T | exonic | DUS3L | nonsynonymous SNV | 19p13.3 | Uncertain significance | DUS3L:NM_001161619:exon1:c.G82A:p.A28T, DUS3L:NM_020175:exon1:c.G82A:p.A28T |
| 19 | 59028254 | 59028254 | C | T | exonic | ZBTB45 | nonsynonymous SNV | 19q13.43 | Uncertain significance | ZBTB45:NM_001316978:exon2:c.G787A:p.A263T, ZBTB45:NM_001316979:exon2:c.G787A:p.A263T, ZBTB45:NM_001316980:exon2:c.G787A:p.A263T, ZBTB45:NM_001316981:exon2:c.G787A:p.A263T, ZBTB45:NM_001316982:exon2:c.G787A:p.A263T, ZBTB45:NM_032792:exon2:c.G787A:p.A263T |
| 2 | 23785220 | 23785220 | G | A | exonic | KLHL29 | nonsynonymous SNV | 2p24.1 | Uncertain significance | KLHL29:NM_052920:exon3:c.G154A:p.G52S |
| 2 | 27167581 | 27167581 | G | A | exonic | DPYSL5 | nonsynonymous SNV | 2p23.3 | Uncertain significance | DPYSL5:NM_001253723:exon12:c.G1498A:p.V500M, DPYSL5:NM_001253724:exon12:c.G1498A:p.V500M, DPYSL5:NM_020134:exon12:c.G1498A:p.V500M |
| 2 | 27279591 | 27279591 | G | A | exonic | AGBL5 | nonsynonymous SNV | 2p23.3 | Uncertain significance | AGBL5:NM_001035507:exon8:c.G1466A:p.R489H, AGBL5:NM_021831:exon8:c.G1466A:p.R489H |
| 2 | 54587672 | 54587672 | A | C | exonic | C2orf73 | nonsynonymous SNV | 2p16.2 | Uncertain significance | C2orf73:NM_001369403:exon4:c.A474C:p.L158F, C2orf73:NM_001100396:exon5:c.A837C:p.L279F, C2orf73:NM_001369401:exon5:c.A663C:p.L221F |
| 2 | 67632137 | 67632137 | A | C | exonic | ETAA1 | nonsynonymous SNV | 2p14 | Uncertain significance | ETAA1:NM_019002:exon5:c.A2323C:p.S775R |
| 2 | 74906749 | 74906749 | C | T | exonic | SEMA4F | nonsynonymous SNV | 2p13.1 | Uncertain significance | SEMA4F:NM_001271661:exon10:c.C1261T:p.P421S, SEMA4F:NM_001271662:exon13:c.C1627T:p.P543S, SEMA4F:NM_004263:exon14:c.C1726T:p.P576S |
| 2 | 75185823 | 75185823 | C | T | exonic | POLE4 | nonsynonymous SNV | 2p12 | Uncertain significance | POLE4:NM_019896:exon1:c.C17T:p.A6V |
| 20 | 17606183 | 17606183 | C | T | exonic | RRBP1 | nonsynonymous SNV | 20p12.1 | Uncertain significance | RRBP1:NM_001365613:exon12:c.G3028A:p.V1010I, RRBP1:NM_004587:exon12:c.G1729A:p.V577I, RRBP1:NM_001042576:exon13:c.G1729A:p.V577I |
| 20 | 47782687 | 47782687 | T | G | exonic | STAU1 | nonsynonymous SNV | 20q13.13 | Uncertain significance | STAU1:NM_001322930:exon2:c.A52C:p.S18R, STAU1:NM_001322932:exon2:c.A52C:p.S18R, STAU1:NM_001322933:exon3:c.A52C:p.S18R, STAU1:NM_017453:exon3:c.A52C:p.S18R, STAU1:NM_001319135:exon4:c.A52C:p.S18R, STAU1:NM_001322929:exon4:c.A52C:p.S18R |
| 22 | 17590230 | 17590230 | C | - | exonic | IL17RA | frameshift deletion | 22q11.1 | - | IL17RA:NM_001289905:exon12:c.2019delC:p.G673fs, IL17RA:NM_014339:exon13:c.2121delC:p.G707fs |
| 22 | 20128949 | 20128949 | G | A | exonic | ZDHHC8 | nonsynonymous SNV | 22q11.21 | Uncertain significance | ZDHHC8:NM_001185024:exon9:c.G1021A:p.A341T, ZDHHC8:NM_013373:exon9:c.G1021A:p.A341T |
| 22 | 32108801 | 32108801 | G | T | exonic | PRR14L | nonsynonymous SNV | 22q12.2 | Uncertain significance | PRR14L:NM_173566:exon4:c.C5024A:p.A1675E |
| 22 | 40662984 | 40662984 | G | C | exonic | TNRC6B | nonsynonymous SNV | 22q13.1 | Uncertain significance | TNRC6B:NM_001162501:exon5:c.G2750C:p.G917A, TNRC6B:NM_015088:exon5:c.G2750C:p.G917A |
| 3 | 111319608 | 111319608 | G | A | exonic | CD96 | nonsynonymous SNV | 3q13.2 | Uncertain significance | CD96:NM_001318889:exon7:c.G934A:p.D312N, CD96:NM_005816:exon7:c.G934A:p.D312N, CD96:NM_198196:exon8:c.G982A:p.D328N |
| 3 | 113672611 | 113672611 | C | T | exonic | ZDHHC23 | nonsynonymous SNV | 3q13.31 | Uncertain significance | ZDHHC23:NM_001320466:exon3:c.C226T:p.R76C, ZDHHC23:NM_001320467:exon3:c.C226T:p.R76C, ZDHHC23:NM_001320468:exon3:c.C226T:p.R76C, ZDHHC23:NM_001363952:exon3:c.C226T:p.R76C, ZDHHC23:NM_173570:exon3:c.C226T:p.R76C |
| 3 | 124438313 | 124438313 | C | T | exonic | KALRN | nonsynonymous SNV | 3q21.2 | Uncertain significance | KALRN:NM_001322993:exon27:c.C3863T:p.T1288M, KALRN:NM_007064:exon27:c.C3866T:p.T1289M, KALRN:NM_001024660:exon60:c.C8957T:p.T2986M |
| 3 | 142281353 | 142281353 | C | G | exonic | ATR | nonsynonymous SNV | 3q23 | Benign | ATR:NM_001184:exon4:c.G891C:p.K297N, ATR:NM_001354579:exon4:c.G891C:p.K297N |
| 3 | 151163828 | 151163828 | G | A | exonic | IGSF10 | nonsynonymous SNV | 3q25.1 | Uncertain significance | IGSF10:NM_178822:exon4:c.C3941T:p.T1314M |
| 3 | 154886561 | 154886561 | A | G | exonic | MME | nonsynonymous SNV | 3q25.2 | Uncertain significance | MME:NM_000902:exon20:c.A1955G:p.N652S, MME:NM_001354642:exon20:c.A1955G:p.N652S, MME:NM_001354643:exon20:c.A1955G:p.N652S, MME:NM_007287:exon20:c.A1955G:p.N652S, MME:NM_007288:exon20:c.A1955G:p.N652S, MME:NM_007289:exon20:c.A1955G:p.N652S |
| 3 | 156867134 | 156867134 | C | G | exonic | CCNL1 | nonsynonymous SNV | 3q25.31 | Uncertain significance | CCNL1:NM_001308185:exon10:c.G1174C:p.A392P, CCNL1:NM_020307:exon10:c.G1174C:p.A392P |
| 3 | 180325561 | 180325561 | C | G | exonic | TTC14 | nonsynonymous SNV | 3q26.33 | Uncertain significance | TTC14:NM_001042601:exon10:c.C1298G:p.P433R |
| 3 | 183824368 | 183824368 | T | A | exonic | HTR3E | nonsynonymous SNV | 3q27.1 | Uncertain significance | HTR3E:NM_001256614:exon7:c.T1336A:p.S446T, HTR3E:NM_198314:exon7:c.T1258A:p.S420T, HTR3E:NM_182589:exon8:c.T1303A:p.S435T, HTR3E:NM_198313:exon8:c.T1213A:p.S405T, HTR3E:NM_001256613:exon9:c.T1258A:p.S420T |
| 3 | 195509045 | 195509045 | C | G | exonic | MUC4 | nonsynonymous SNV | 3q29 | Likely benign | MUC4:NM_018406:exon2:c.G9406C:p.A3136P |
| 3 | 195513180 | 195513180 | C | G | exonic | MUC4 | nonsynonymous SNV | 3q29 | Benign | MUC4:NM_018406:exon2:c.G5271C:p.Q1757H |
| 3 | 195513214 | 195513214 | A | G | exonic | MUC4 | nonsynonymous SNV | 3q29 | Benign | MUC4:NM_018406:exon2:c.T5237C:p.L1746P |
| 3 | 195513227 | 195513227 | C | T | exonic | MUC4 | nonsynonymous SNV | 3q29 | Uncertain significance | MUC4:NM_018406:exon2:c.G5224A:p.A1742T |
| 3 | 20025292 | 20025292 | A | G | exonic | RAB5A | nonsynonymous SNV | 3p24.3 | Uncertain significance | RAB5A:NM_001292048:exon6:c.A583G:p.R195G, RAB5A:NM_004162:exon6:c.A625G:p.R209G |
| 3 | 20216229 | 20216229 | A | C | exonic | SGO1 | nonsynonymous SNV | 3p24.3 | Uncertain significance | SGO1:NM_001012409:exon6:c.T794G:p.F265C, SGO1:NM_001012410:exon6:c.T794G:p.F265C, SGO1:NM_001199251:exon6:c.T794G:p.F265C, SGO1:NM_001199252:exon6:c.T794G:p.F265C |
| 3 | 36931380 | 36931380 | C | T | exonic | TRANK1 | nonsynonymous SNV | 3p22.2 | Uncertain significance | TRANK1:NM_014831:exon7:c.G715A:p.G239R, TRANK1:NM_001329998:exon8:c.G847A:p.G283R |
| 3 | 38924778 | 38924778 | G | C | exonic | SCN11A | nonsynonymous SNV | 3p22.2 | Likely pathogenic | SCN11A:NM_014139:exon18:c.C3165G:p.H1055Q, SCN11A:NM_001349253:exon22:c.C3165G:p.H1055Q |
| 3 | 39178807 | 39178807 | C | T | exonic | TTC21A | nonsynonymous SNV | 3p22.2 | Uncertain significance | TTC21A:NM_001105513:exon24:c.C3256T:p.R1086W, TTC21A:NM_001366899:exon25:c.C3403T:p.R1135W, TTC21A:NM_001366900:exon25:c.C3379T:p.R1127W, TTC21A:NM_145755:exon25:c.C3400T:p.R1134W |
| 3 | 39225880 | 39225880 | G | A | exonic | XIRP1 | nonsynonymous SNV | 3p22.2 | Uncertain significance | XIRP1:NM_001351377:exon2:c.C1106T:p.P369L, XIRP1:NM_194293:exon2:c.C5057T:p.P1686L |
| 3 | 40529209 | 40529209 | A | G | exonic | ZNF619 | nonsynonymous SNV | 3p22.1 | Uncertain significance | ZNF619:NM_001145083:exon4:c.A1076G:p.N359S, ZNF619:NM_001145093:exon5:c.A1208G:p.N403S, ZNF619:NM_001145082:exon6:c.A1328G:p.N443S, ZNF619:NM_001145094:exon6:c.A1181G:p.N394S, ZNF619:NM_001363277:exon6:c.A1034G:p.N345S, ZNF619:NM_173656:exon6:c.A1160G:p.N387S |
| 3 | 47957741 | 47957741 | T | C | exonic | MAP4 | nonsynonymous SNV | 3p21.31 | Uncertain significance | MAP4:NM_001134364:exon7:c.A1576G:p.T526A, MAP4:NM_002375:exon7:c.A1576G:p.T526A |
| 3 | 48627963 | 48627963 | G | A | exonic | COL7A1 | nonsynonymous SNV | 3p21.31 | Uncertain significance | COL7A1:NM_000094:exon14:c.C1835T:p.T612M |
| 3 | 48681704 | 48681704 | C | T | exonic | CELSR3 | nonsynonymous SNV | 3p21.31 | Uncertain significance | CELSR3:NM_001407:exon27:c.G8110A:p.A2704T |
| 3 | 52410004 | 52410004 | G | A | exonic | DNAH1 | nonsynonymous SNV | 3p21.1 | Uncertain significance | DNAH1:NM_015512:exon46:c.G7193A:p.R2398H |
| 3 | 52821210 | 52821210 | C | T | exonic | ITIH1 | nonsynonymous SNV | 3p21.1 | Uncertain significance | ITIH1:NM_001166435:exon11:c.C1031T:p.P344L, ITIH1:NM_001166436:exon11:c.C1031T:p.P344L, ITIH1:NM_001166434:exon13:c.C1469T:p.P490L, ITIH1:NM_002215:exon15:c.C1895T:p.P632L |
| 3 | 93779907 | 93779907 | - | A | exonic | DHFR2 | frameshift insertion | 3q11.1 | - | DHFR2:NM_001195643:exon2:c.448dupT:p.S150fs, DHFR2:NM_176815:exon2:c.448dupT:p.S150fs |
| 4 | 152203448 | 152203448 | T | G | exonic | PRSS48 | nonsynonymous SNV | 4q31.3 | Uncertain significance | PRSS48:NM_183375:exon3:c.T364G:p.S122A |
| 4 | 2758009 | 2758009 | C | T | exonic | TNIP2 | nonsynonymous SNV | 4p16.3 | Uncertain significance | TNIP2:NM_001292016:exon1:c.G8A:p.R3Q, TNIP2:NM_024309:exon1:c.G8A:p.R3Q |
| 5 | 120022309 | 120022309 | A | T | exonic | PRR16 | nonsynonymous SNV | 5q23.1 | Uncertain significance | PRR16:NM_001300783:exon2:c.A820T:p.S274C, PRR16:NM_001308087:exon2:c.A610T:p.S204C, PRR16:NM_016644:exon3:c.A751T:p.S251C, PRR16:NM_001308088:exon4:c.A610T:p.S204C |
| 5 | 140433015 | 140433015 | T | C | exonic | PCDHB1 | nonsynonymous SNV | 5q31.3 | Uncertain significance | PCDHB1:NM_013340:exon1:c.T1960C:p.S654P |
| 5 | 140568922 | 140568922 | C | A | exonic | PCDHB9 | unknown | 5q31.3 | Uncertain significance | UNKNOWN |
| 5 | 140768269 | 140768269 | T | G | exonic | PCDHGB4 | nonsynonymous SNV | 5q31.3 | Uncertain significance | PCDHGB4:NM_003736:exon1:c.T818G:p.V273G, PCDHGB4:NM_032098:exon1:c.T818G:p.V273G |
| 5 | 140795208 | 140795208 | - | A | exonic | PCDHGA10 | frameshift insertion | 5q31.3 | - | PCDHGA10:NM_032090:exon1:c.2467dupA:p.V822fs |
| 5 | 71015151 | 71015151 | C | A | exonic | CARTPT | nonsynonymous SNV | 5q13.2 | Uncertain significance | CARTPT:NM_004291:exon1:c.C31A:p.L11I |
| 5 | 73183505 | 73183505 | G | T | splicing | ARHGEF28 | splicing | 5q13.2 | - | NA |
| 6 | 41655542 | 41655542 | C | T | exonic | TFEB | nonsynonymous SNV | 6p21.1 | Uncertain significance | TFEB:NM_001271943:exon5:c.G421A:p.E141K, TFEB:NM_001167827:exon6:c.G718A:p.E240K, TFEB:NM_001271944:exon6:c.G676A:p.E226K, TFEB:NM_001271945:exon6:c.G676A:p.E226K, TFEB:NM_007162:exon7:c.G676A:p.E226K |
| 7 | 112407719 | 112407719 | C | T | exonic | TMEM168 | nonsynonymous SNV | 7q31.1 | Uncertain significance | TMEM168:NM_022484:exon5:c.G1627A:p.V543I, TMEM168:NM_001287497:exon6:c.G1627A:p.V543I |
| 7 | 6464390 | 6464390 | G | C | exonic | DAGLB | nonsynonymous SNV | 7p22.1 | Uncertain significance | DAGLB:NM_001142936:exon6:c.C746G:p.S249C, DAGLB:NM_139179:exon8:c.C1133G:p.S378C |
| 7 | 6465651 | 6465651 | T | C | exonic | DAGLB | nonsynonymous SNV | 7p22.1 | Uncertain significance | DAGLB:NM_001142936:exon5:c.A637G:p.R213G, DAGLB:NM_139179:exon7:c.A1024G:p.R342G |
| 7 | 889631 | 889631 | G | A | exonic | SUN1 | nonsynonymous SNV | 7p22.3 | - | SUN1:NM_001367639:exon5:c.G163A:p.G55S, SUN1:NM_001367645:exon6:c.G580A:p.G194S, SUN1:NM_001367648:exon6:c.G580A:p.G194S, SUN1:NM_001367667:exon6:c.G580A:p.G194S, SUN1:NM_001367679:exon6:c.G580A:p.G194S, SUN1:NM_001367689:exon6:c.G580A:p.G194S, SUN1:NM_001367695:exon6:c.G541A:p.G181S, SUN1:NM_001367638:exon7:c.G730A:p.G244S, SUN1:NM_001367647:exon7:c.G664A:p.G222S, SUN1:NM_001367649:exon7:c.G664A:p.G222S, SUN1:NM_001367664:exon7:c.G730A:p.G244S, SUN1:NM_001367668:exon7:c.G664A:p.G222S, SUN1:NM_001367684:exon7:c.G778A:p.G260S, SUN1:NM_001367685:exon7:c.G730A:p.G244S, SUN1:NM_001367696:exon7:c.G730A:p.G244S, SUN1:NM_001367702:exon7:c.G730A:p.G244S, SUN1:NM_001367704:exon7:c.G730A:p.G244S, SUN1:NM_001367636:exon8:c.G862A:p.G288S, SUN1:NM_001367643:exon8:c.G814A:p.G272S, SUN1:NM_001367651:exon8:c.G1033A:p.G345S, SUN1:NM_001367655:exon8:c.G862A:p.G288S, SUN1:NM_001367666:exon8:c.G862A:p.G288S, SUN1:NM_001367677:exon8:c.G928A:p.G310S, SUN1:NM_001367691:exon8:c.G862A:p.G288S, SUN1:NM_001367692:exon8:c.G928A:p.G310S, SUN1:NM_001367693:exon8:c.G814A:p.G272S, SUN1:NM_001367697:exon8:c.G814A:p.G272S, SUN1:NM_001367700:exon8:c.G928A:p.G310S, SUN1:NM_001367678:exon9:c.G1012A:p.G338S, SUN1:NM_001367687:exon9:c.G1012A:p.G338S, SUN1:NM_001367690:exon9:c.G1012A:p.G338S, SUN1:NM_001367699:exon9:c.G1012A:p.G338S, SUN1:NM_001367703:exon9:c.G1012A:p.G338S, SUN1:NM_001367705:exon9:c.G1012A:p.G338S |
| 8 | 33406952 | 33406952 | G | A | exonic | RNF122 | nonsynonymous SNV | 8p12 | Uncertain significance | RNF122:NM_024787:exon5:c.C329T:p.P110L |
| 9 | 114520363 | 114520363 | C | A | splicing | SHOC1 | splicing | 9q31.3 | - | NA |
| 9 | 116356458 | 116356458 | G | A | exonic | RGS3 | nonsynonymous SNV | 9q32 | Uncertain significance | RGS3:NM_144489:exon1:c.G259A:p.V87I |
| 9 | 123676565 | 123676565 | A | G | exonic | TRAF1 | nonsynonymous SNV | 9q33.2 | Uncertain significance | TRAF1:NM_005658:exon4:c.T242C:p.V81A, TRAF1:NM_001190945:exon5:c.T242C:p.V81A |
| 9 | 130191194 | 130191194 | C | T | exonic | ZNF79 | nonsynonymous SNV | 9q33.3 | Uncertain significance | ZNF79:NM_001286696:exon2:c.C28T:p.P10S, ZNF79:NM_007135:exon2:c.C100T:p.P34S, ZNF79:NM_001286697:exon3:c.C28T:p.P10S, ZNF79:NM_001322260:exon3:c.C28T:p.P10S |
| 9 | 130422343 | 130422343 | C | T | exonic | STXBP1 | nonsynonymous SNV | 9q34.11 | Uncertain significance | STXBP1:NM_001032221:exon5:c.C281T:p.P94L, STXBP1:NM_003165:exon5:c.C281T:p.P94L |
| 9 | 131749872 | 131749872 | A | T | exonic | NUP188 | nonsynonymous SNV | 9q34.11 | Uncertain significance | NUP188:NM_015354:exon23:c.A2269T:p.T757S |
| 9 | 135537982 | 135537982 | A | T | exonic | DDX31 | nonsynonymous SNV | 9q34.13 | Uncertain significance | DDX31:NM_001322340:exon2:c.T491A:p.V164D, DDX31:NM_001322343:exon2:c.T104A:p.V35D, DDX31:NM_001322344:exon2:c.T491A:p.V164D, DDX31:NM_022779:exon2:c.T491A:p.V164D, DDX31:NM_138620:exon2:c.T491A:p.V164D, DDX31:NM_001322341:exon3:c.T203A:p.V68D |
| 9 | 139360847 | 139360847 | C | T | exonic | SEC16A | nonsynonymous SNV | 9q34.3 | Uncertain significance | SEC16A:NM_001276418:exon6:c.G3997A:p.D1333N, SEC16A:NM_014866:exon7:c.G3997A:p.D1333N |
| 9 | 35403758 | 35403758 | G | A | exonic | UNC13B | nonsynonymous SNV | 9p13.3 | Uncertain significance | UNC13B:NM_001371188:exon30:c.G3394A:p.E1132K, UNC13B:NM_001371187:exon33:c.G5701A:p.E1901K, UNC13B:NM_001371186:exon39:c.G4501A:p.E1501K, UNC13B:NM_006377:exon39:c.G4504A:p.E1502K, UNC13B:NM_001330653:exon40:c.G4561A:p.E1521K, UNC13B:NM_001371189:exon40:c.G12751A:p.E4251K |
| 9 | 43822749 | 43822749 | G | A | exonic | CNTNAP3B | nonsynonymous SNV | 9p11.2 | Uncertain significance | CNTNAP3B:NM_001201380:exon8:c.G1303A:p.A435T |
| 9 | 70483186 | 70483186 | A | G | splicing | CBWD5 | splicing | 9q21.11 | - | NA |
| X | 107908766 | 107908766 | A | G | exonic | COL4A5 | nonsynonymous SNV | Xq22.3 | Uncertain significance | COL4A5:NM_000495:exon38:c.A3403G:p.I1135V |
| X | 108779154 | 108779154 | G | C | exonic | NXT2 | nonsynonymous SNV | Xq23 | Uncertain significance | NXT2:NM_018698:exon1:c.G43C:p.G15R |
| X | 50653913 | 50653913 | C | G | exonic | BMP15 | nonsynonymous SNV | Xp11.22 | Uncertain significance | BMP15:NM_005448:exon1:c.C130G:p.L44V |
| X | 69479026 | 69479026 | C | G | exonic | P2RY4 | nonsynonymous SNV | Xq13.1 | Uncertain significance | P2RY4:NM_002565:exon1:c.G449C:p.R150P |
