# Supplementary material for: Haematological malignancies in relatives of patients affected with myeloproliferative neoplasms
Source: EJHaem. 2022 Mar 24;3(2):475–9. doi: 10.1002/jha2.425 (PMC9176120; doi:10.1002/jha2.425)
Supplement: Supplementary file 4 — Supporting Information [file JHA2-3-475-s001.docx]

Supplementary table 4 – Results of WES analysis showing the 7 structural variants detected in both individuals of family #126

| Chr_1 | Pos_1 | Dir_1 | Chr_2 | Pos_2 | Dir_2 | Inserted_Seq | Variant_Type | Gene_1 | Gene_2 | Exon_1 | Exon_2 |
| --- | --- | --- | --- | --- | --- | --- | --- | --- | --- | --- | --- |
| 5 | 68471095 | + | 5 | 68471150 | - | --- | deletion | CCNB1 | CCNB1 | --- | --- |
| 6 | 129920298 | + | 6 | 129920322 | - | --- | deletion | ARHGAP18 | ARHGAP18 | --- | --- |
| 9 | 136637192 | - | 9 | 136637222 | + | --- | tandem_duplication | VAV2 | VAV2 | --- | --- |
| 16 | 1500694 | + | 16 | 1500933 | - | --- | deletion | CLCN7 | CLCN7 | --- | --- |
| 19 | 45719295 | + | 19 | 45719350 | - | --- | deletion | EXOC3L2 | EXOC3L2 | --- | EXOC3L2 |
| 19 | 45719299 | + | 19 | 45719354 | - | --- | deletion | EXOC3L2 | EXOC3L2 | --- | EXOC3L2 |
| 19 | 45911321 | - | 19 | 45911340 | + | --- | tandem_duplication | CD3EAP;ERCC1 | CD3EAP;ERCC1 | ERCC1 | ERCC1 |
