# Supplementary material for: Haematological malignancies in relatives of patients affected with myeloproliferative neoplasms
Source: EJHaem. 2022 Mar 24;3(2):475–9. doi: 10.1002/jha2.425 (PMC9176120; doi:10.1002/jha2.425)
Supplement: Supplementary file 5 — Supporting Information [file JHA2-3-475-s003.docx]

| Chr_1 | Pos_1 | Dir_1 | Chr_2 | Pos_2 | Dir_2 | Inserted_Seq | Variant_Type | Gene_1 | Gene_2 | Exon_1 | Exon_2 |
| --- | --- | --- | --- | --- | --- | --- | --- | --- | --- | --- | --- |
| 1 | 150315760 | - | 1 | 150315790 | + | --- | tandem_duplication | PRPF3 | PRPF3 | --- | PRPF3 |
| 2 | 115920072 | + | 2 | 118981692 | + | --- | inversion | DPP10 | --- | --- | --- |
| 10 | 89652823 | + | 10 | 89653723 | - | --- | deletion | PTEN | PTEN | --- | --- |
| 15 | 41851540 | + | 15 | 41853322 | - | AGTCCGCCGCCG | deletion | TYRO3 | TYRO3 | TYRO3 | --- |
| 15 | 41866013 | + | 15 | 41870084 | - | --- | deletion | TYRO3 | TYRO3 | TYRO3 | TYRO3 |
| 16 | 1500694 | + | 16 | 1500933 | - | --- | deletion | CLCN7 | CLCN7 | --- | --- |
| X | 70341123 | - | X | 70341136 | + | --- | tandem_duplication | MED12 | MED12 | --- | --- |

Supplementary table 5 - Results of WES analysis showing the 7 structural variants detected in both individuals of family #127
